# PARP1-cGAS-NF-κB pathway of proinflammatory macrophage activation by extracellular vesicles released during *Trypanosoma cruzi* infection and Chagas disease

**DOI:** 10.1371/journal.ppat.1008474

**Published:** 2020-04-21

**Authors:** Subhadip Choudhuri, Nisha Jain Garg

**Affiliations:** 1 Department of Microbiology and Immunology, University of Texas Medical Branch (UTMB), Galveston, Texas, United States of America; 2 Institute for Human Infections and Immunity (IHII), UTMB, Galveston, Texas, United States of America; National Institute of Health, UNITED STATES

## Abstract

*Trypanosoma cruzi* (*T*. *cruzi*) is the etiological agent of Chagas cardiomyopathy. In the present study, we investigated the role of extracellular vesicles (Ev) in shaping the macrophage (Mφ) response in progressive Chagas disease (CD). We purified *T*. *cruzi* Ev (*Tc*Ev) from axenic parasite cultures, and *T*. *cruzi*-induced Ev (TEv) from the supernatants of infected cells and plasma of acutely and chronically infected wild-type and *Parp1*^*-/-*^ mice. Cultured (Raw 264.7) and bone-marrow Mφ responded to *Tc*EV and TEv with a profound increase in the expression and release of TNF-α, IL-6, and IL-1β cytokines. TEv produced by both immune (Mφ) and non-immune (muscle) cells were proinflammatory. Chemical inhibition or genetic deletion of PARP1 (a DNA repair enzyme) significantly depressed the TEv-induced transcriptional and translational activation of proinflammatory Mφ response. Oxidized DNA encapsulated by TEv was necessary for PARP1-dependent proinflammatory Mφ response. Inhibition studies suggested that DNA-sensing innate immune receptors (cGAS>>TLR9) synergized with PARP1 in signaling the NFκB activation, and inhibition of PARP1 and cGAS resulted in >80% inhibition of TEv-induced NFκB activity. Histochemical studies showed intense inflammatory infiltrate associated with profound increase in CD11b^+^CD68^+^TNF-α^+^ Mφ in the myocardium of CD wild-type mice. In comparison, chronically infected *Parp1*^-/-^ mice exhibited low-to-moderate tissue inflammation, >80% decline in myocardial infiltration of TNF-α^+^ Mφ, and no change in immunoregulatory IL-10^+^ Mφ. We conclude that oxidized DNA released with TEv signal the PARP1-cGAS-NF-κB pathway of proinflammatory Mφ activation and worsens the chronic inflammatory pathology in CD. Small molecule antagonists of PARP1-cGAS signaling pathway would potentially be useful in reprogramming the Mφ activation and controlling the chronic inflammation in CD.

## Author summary

Chagas disease, caused by *Trypanosoma cruzi (T*. *cruzi)* parasite, is a life-threatening debilitating illness. Tissue inflammation is a hallmark of clinical form of Chagas dilated cardiomyopathy. Recent studies show that cell membranes shed extracellular vesicles (Ev) that carry DNA, proteins, and lipids of host and pathogen origin. In this study, we show that Ev released by *T*. *cruzi*, *T*. *cruzi*-infected cells and in plasma of Chagas mice (vs. controls) carry parasite and host DNA that is oxidized. When captured by macrophages, the Ev-carried oxidized DNA molecules were recognized by cytosolic DNA sensors cGAS and PARP1, and subsequently, signaled NF-κB-mediated proinflammatory cytokine production. Chemical or genetic inhibition of PARP1 resulted in significant decrease in Ev-induced inflammatory response of macrophages and cardiac inflammation in Chagas mice. We propose that chemical inhibitors of PARP1 offer a potential therapeutic target in arresting chronic inflammation in Chagas disease through modulation of the macrophage proinflammatory signaling.

## Introduction

Chagas disease (CD) is an inflammatory, dilated cardiomyopathy caused by flagellated protozoa *Trypanosoma cruzi* (*T*. *cruzi*). The infection may be acquired through the vector-borne or transplacental routes, transfusion of contaminated blood components, or from a transplanted organ of an infected donor [1]. Exposure to pathogen results in acute parasitemia associated brief illness that in most cases is resolved without clinical intervention. Several years later, ~30% of the infected patients progress into clinically symptomatic, chronic CD when they display cardiac insufficiency due to tissue fibrosis, ventricular dilation, and arrhythmia. Chagas cardiomyopathy continues to result in a loss of 2.74 million disability-adjusted life years, and 15,000 deaths due to heart failure per year [2].

Macrophages (Mφ) are the innate immune cells that play a critical role in modulating the host response to *T*. *cruzi* infection [3]. Classically activated Mφ, differentiated through the IL-12/IFN-γ axis, play a critical role in control of *T*. *cruzi* infection [4]. It has been documented that parasite killing is triggered in Mφ by autocrine TNF-α secretion. As antigen presenting cells, Mφ also contribute to the activation of Th1 CD4^+^T cells and cytolytic CD8^+^T cells that are essential for killing the intracellular, replicative form of *T*. *cruzi* [5]. A significant presence of Mφ is also noted during the progression of chronic Chagas disease. Stimulus for Mφ proliferation and activation and the role these cells may play in chronic CD is not fully understood [2, 6].

Extracellular vesicles (Ev) are small vesicles harboring ligands, receptors, active lipids or RNA/DNA from the cell of their origin [7]. In pathological conditions, a stimulus that triggers Ev formation regulates the selective sorting of constituents and composition of Ev, and consequently, the biological information that they transfer. Recently, it was shown that Ev produced by *T*. *cruzi* trypomastigotes (infective form) fuse to host cell membranes and promote Ev release from THP-1 Mφ [8, 9]. We have found that human peripheral blood mononuclear cells (PBMC) incubated with *T*. *cruzi* secreted Ev and the latter elicited a proinflammatory gene expression profile in human THP-1 Mφ [3]. A proinflammatory cytokine response was also noted when THP-1 Mφ were incubated with Ev isolated from peripheral blood of CD patients [10]. These findings indicate that exposure to *T*. *cruzi* influences the host cell Ev release, and the Ev have an impact on the surrounding infected or injured tissue [10]. The mechanism(s) of Ev-dependent Mφ activation and whether this is helpful or harmful to the infected host is not studied.

Poly(ADP-ribose) polymerase 1 (PARP1) is a 113-kDa protein (89-kDa active form) that belongs to the PARP family of seven known and ten putative members, and it accounts for >85% of the PARP activity in cellular systems [11]. PARP1 catalyzes the cleavage of NAD^+^ into nicotinamide and ADP- ribose and uses the latter to synthesize poly (ADP-ribose) (PAR) polymers. The basal level activation of PARP1 by mild genotoxic stimuli causes PARylation of histone proteins (e.g. H1 and H2B) that mediates relaxation of the chromatin superstructure and recruitment of DNA-break repair enzymes, resulting in DNA repair and cell survival [12, 13]. PARP1, by direct binding to or PARylation of enhancers and promoters, can also function as a transcriptional co-activator and modulate the expression of self and many other genes. However, excessive activation of PARP1 has been considered pathologic, and linked to a number of cancers, central nervous system disorders, and heart failure [12, 14]. Accordingly, in recent years, significant efforts have been devoted to the development and testing of PARP1 targeted therapies. We have found that PARP1/PAR enhanced the mitochondrial production of reactive oxygen species (mtROS) and ROS-dependent NF-κB activation in cardiomyocytes infected by *T*. *cruzi*, and over-expression of PARP1/PAR might be of pathologic significance in chronic Chagas disease [15].

In this study, we aimed to determine the role of Ev released during *T*. *cruzi* infection and chronic CD in shaping the Mφ response and investigated the signaling mechanisms that mediate Ev-dependent Mφ activation. For this, we isolated Ev from media of cultured *T*. *cruzi* trypomastigotes, supernatants of immune and non-immune cells infected with *T*. *cruzi*, and plasma of acutely and chronically infected mice. We used cultured Mφ and primary Mφ isolated from bone marrow (BM) of wild type (WT) and *Parp1*^-/-^ mice and employed classical approaches to evaluate the Ev-PARP1 signaling of Mφ activation. We also fractionated the Ev and used a variety of selective inhibitors to determine the role of DNA- and protein-recognizing innate immune receptors in Ev-PARP1 signaling of Mφ response in Chagas disease. We discuss the benefits of PARP1-targeted therapies in controlling the inflammatory pathology in CD.

## Results

### Cytokine profile of macrophages elicited by *T*. *cruzi-*induced extracellular vesicles (TEv)

We first determined if *T*. *cruzi* infection promotes the release of Ev capable of programming Mφ response. For this, we isolated *T*. *cruzi*-induced Ev (TEv) from supernatants of Raw 264.7 Mφ at 24 h, 48 h, and 72 h post-infection. A new batch of Mφ were incubated for 48 h with TEv and supernatants were analyzed by an ELISA. TEv isolated at 72 h elicited the maximal activation of TNF-α release, while TEv isolated at 24 h were minimally active in inducing Mφ activation (S1A Fig). No effect of presence or absence of heat-inactivated FBS on TEv-induced Mφ release of TNF-α was observed (S1B Fig). Based on these observations, further experiments were conducted using TEv isolated at 72 h post-incubation.

**Fig 1:**
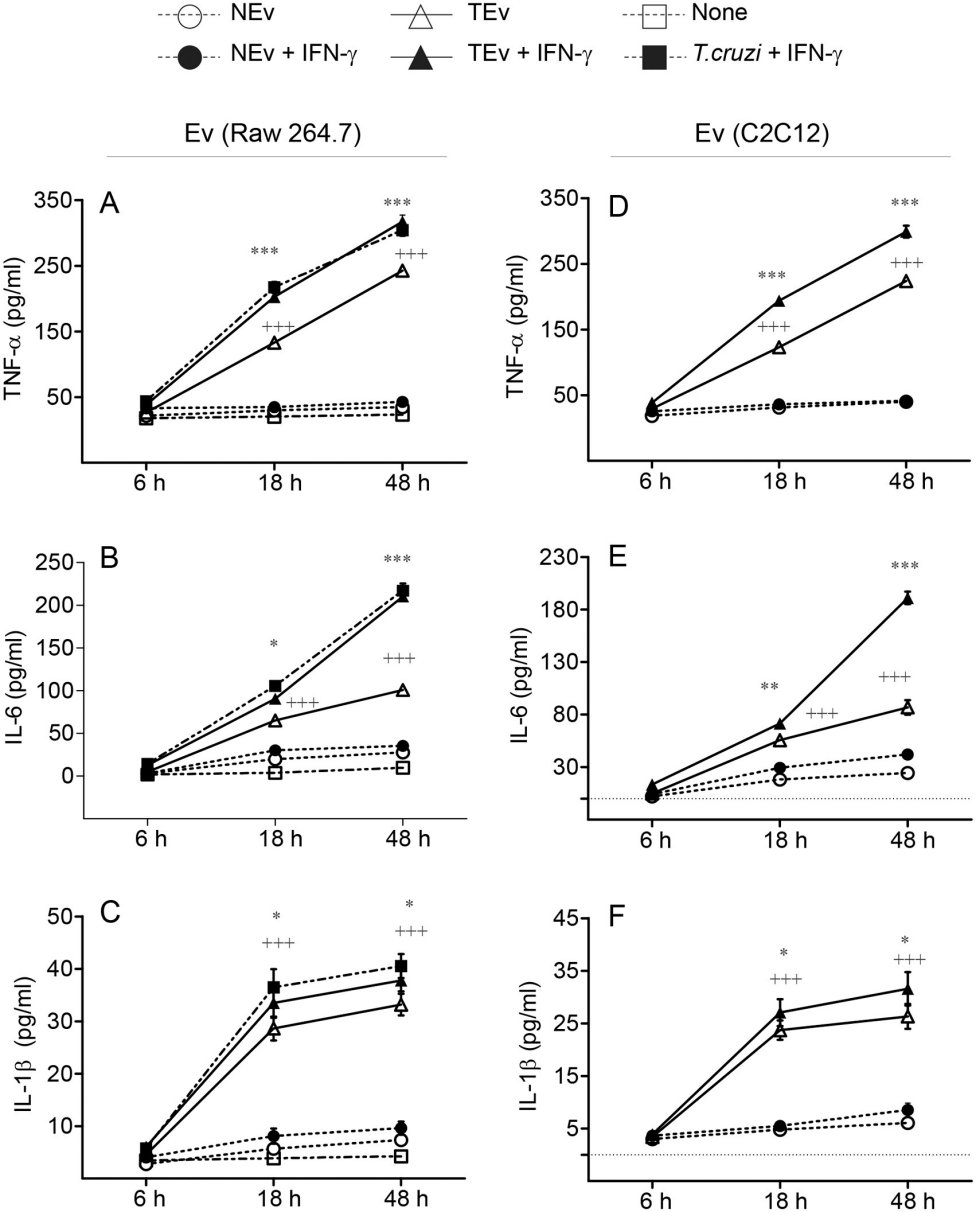
*T*. *cruzi* induced Ev elicit proinflammatory cytokines release in macrophages. Raw 264.7 Mφ and C2C12 muscle cells were incubated with media only or *T*. *cruzi* (cell: parasite ratio, 1: 3) for 72 h. Extracellular vesicles (Ev) released in supernatants were isolated as described in Materials and Methods. Next, cultured Mφ were incubated with Ev isolated from supernatants of Raw Mφ **(A-C)** and C2C12 cells **(D-F)** for 0–48 h (± 20 ng/mL IFN-γ) and an ELISA was performed to measure the release of TNF-α ***(A&D)***, IL-6 ***(B&E)***, and IL-1β ***(C&F)*** cytokines. Raw Mφ infected with *T*. *cruzi* or incubated with media alone were used as positive and negative controls, respectively. NEv: Ev isolated from non-infected cells; TEv: Ev isolated from supernatants of *T*. *cruzi-*infected cells. Data are representative of ≥ 2 independent experiments (three biological replicates per treatment, duplicate observations per sample) and plotted as mean value ± SD. Significance is annotated as ^+^ TEv vs. NEv and * TEv+IFN-γ vs TEv, and *p* values of ≤ 0.05, ≤ 0.01, and ≤ 0.001 are marked with one, two, and three symbol characters, respectively.

To evaluate in detail the effect of TEv on Mφ, we incubated Raw 264.7 Mφ or C2C12 muscle cells with *T*. *cruzi*, and supernatants were processed to isolate *T*. *cruzi*-induced Ev (TEv) at 72 h. Then, we treated cultured Mφ for 0–48 h with TEv and monitored the TEv-induced cytokines release by an ELISA. Mφ incubated with normal Ev (NEv) isolated from non-infected cells were used as controls. Cultured Mφ, incubated with TEv_Raw_ exhibited no increase in cytokines release at 6 h. However, at 18 h and 48 h post-incubation, TEv_Raw_ (vs. NEv_Raw_) elicited 3.5–6.0-fold, 2.3–2.7-fold, and 3.5–4.0- fold increase in the release of TNF-α, IL-6, and IL-1β, respectively, and maximal cytokines’ release was observed at 48 h post-incubation (Fig 1A–1C, all, ^+^p<0.001). Co-incubation with IFN-γ further increased the TEv_Raw_-induced TNF-α, IL-6, and IL-1β release by 31–52%, 40–110%, and 15-18%, respectively, in Mφ (Fig 1A–1C, all, *p<0.05). The extent of cytokines’ production in Mφ treated with TEv_Raw_+IFN-γ for 18 h and 48 h was similar to that noted in Mφ incubated with live *T*. *cruzi* (cell: parasite ratio, 1:3) and 20 ng/mL IFN-γ (Fig 1A–1C). No significant changes in cytokine’s release were observed in NEv-treated Mφ.

The Ev derived from infected muscle cells that are also the main target of *T*. *cruzi* infection elicited a similar pattern of cytokines’ release, as described above. Raw 264.7 Mφ incubated with TEv_C2C12_ (vs. NEv_C2C12_) exhibited 3.0–4.7-fold, 2.1–2.6-fold, and 3.4–3.9-fold increase in TNF-α, IL-6, and IL-1β release, respectively, during 18–48 h post-incubation (Fig 1D–1F, all, ^+^p<0.001). Co-incubation with IFN-γ further increased the TEv_C2C12_-induced TNF-α, IL-6, and IL-1β release by 30–55%, 30–120%, and 15–20% respectively, during 18–48 h post-incubation (Fig 1D–1F, all, *p<0.05). Moreover, *Tc*Ev obtained from axenic *T*. *cruzi* trypomastigote cultures also elicited Mφ response, evidenced by 3.2–5.2-fold and 3.5–3.8-fold increase in TNF-α, and IL-6 release, respectively, at 18 h and 48 h (S2 Fig, & p< 0.001). Together, the results presented in Fig 1, S1 and S2 Figs suggest that *T*. *cruzi* releases Ev (*Tc*Ev) and *T*. *cruzi* induces release of TEv from innate immune cells and non-immune muscle cells, and these Ev elicit a proinflammatory cytokines’ response in cultured macrophages.

### PARP1 signals cytokine gene expression in Mφ incubated with *T*. *cruzi-*induced Ev

**Fig 2:**
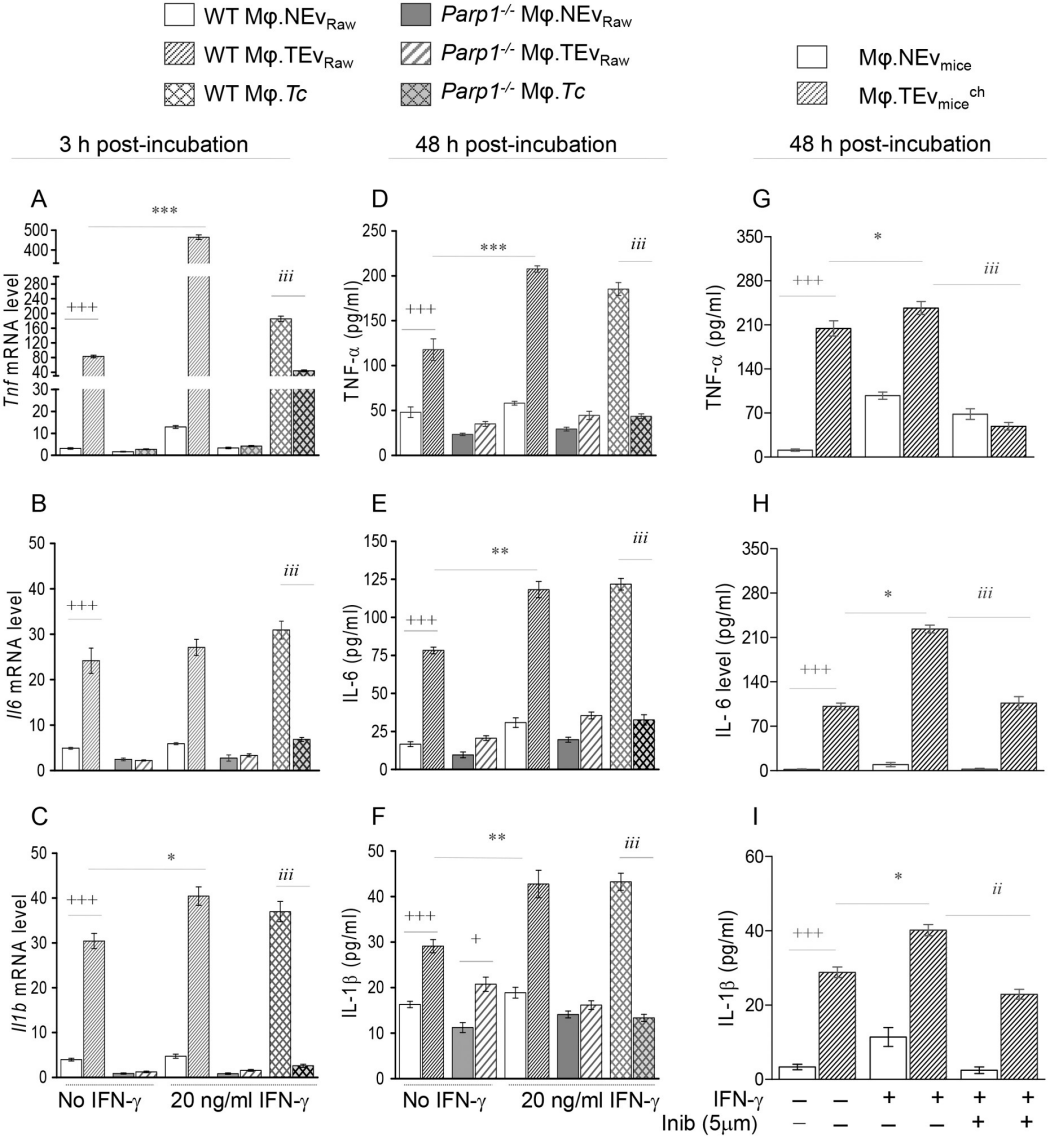
**(A-F) *Parp1***^***-/-***^ **primary Mφ exhibit decreased cytokines’ expression in response to TEv stimulation**. Supernatants of Raw 264.7 Mφ incubated with media only or *T*. *cruzi* were used to purify NEv_RAW_ and TEv_RAW_, respectively. Primary bone-marrow Mφ (WT or *Parp1*^-/-^) were incubated with Ev (± 20 ng/mL IFN-γ) for 3 h and 48 h. ***(A-C)*** Real time RT-qPCR analysis of mRNA levels for cytokine encoding genes at 3 h post-incubation. ***(D-F)*** ELISA determination of cytokines release at 48 h post-incubation. Primary Mφ incubated with *T*. *cruzi* (cell: parasite ratio, 1:3) and IFN-γ were used as controls. **(G-I) Immune characteristics of Ev produced during the development of CD (**± **PARP1 inhibitor)**. B6129S/J WT mice (n = 10 per group) were infected with *T*. *cruzi* (10,000 parasites per mouse). Plasma of normal and chronically infected (150 days post-infection) mice were used to isolate NEv_mice_ and TEv_mice_^ch^, respectively. Next, Mφ were incubated with murine plasma Ev (± 20 ng/ml IFN-γ and 5 μM iniparib) for 48 h, and an ELISA was performed to determine the TNF-α ***(G)***, IL-6 ***(H)***, and IL-1β ***(I)*** release in supernatants. Data are representative of ≥ 2 independent experiments (two biological replicates per treatment and triplicate observations per sample for RT-qPCR analysis; and three biological replicates per treatment and duplicate observations per sample for ELISA). Data are plotted as mean value ± SD. Significance is annotated as ^+^ NEv vs. TEv, * TE_V_ vs. TEv+IFN-γ, and ^*i*^ WT.*Tc* vs. *Parp1*^-/-^.*Tc* and or effect of iniparib on TEv+IFN-γ; and p values of ≤ 0.05, ≤ 0.01, and ≤ 0.001 are presented by one, two, and three symbol characters, respectively. Horizontal bars show the compared groups.

We have previously shown that a PARP1 inhibitor (PJ34) repressed the gene expression for TNF-α and IL-1β cytokines in cardiomyocytes infected by *T*. *cruzi* [14]. PARP1 is found in both cytosolic and nuclear compartments and may influence the proinflammatory cytokine response of Mφ at the gene expression level and/or translational/post- translational level. To sort this out, we isolated primary BM-Mφ from WT and *Parp1*^-/-^ mice, and incubated with TEv_Raw_ and NEv_Raw_ for 3 h, 18 h, and 48 h. WT BM-Mφ responded to TEv_Raw_ by a potent cytokine gene expression, evidenced by 25-fold, 3.9-fold, and 6.6-fold increase in the *Tnf*, *Il6*, and *Il1b* mRNA levels, respectively, at 3 h post-incubation (vs. NEv, Fig 2A–2C, ^+^p<0.001). The TEv-induced cytokines’ gene expression in WT BM-Mφ was not further enhanced at 18 h (S3A–S3C Fig). Co-incubation with IFN-γ led to 4.6-fold further increase in *Tnf* mRNA level (vs. TEv_Raw_, Fig 2A, *p<0.001), though IFN-γ did not promote a significant increase in TEv-induced *Il-6* and *Il1b* mRNA levels in WT BM-Mφ at 3 h or 18 h (Fig 2B & 2C; S3B & S3C Fig). In contrast, *Parp1*^-/-^ Mφ exhibited low levels of cytokines’ gene expression when incubated with TEv (± IFN-γ) for 3 h (Fig 2A–2C) and 18 h (S3A–S3C Fig). When infected with *T*. *cruzi* trypomastigotes (+IFN-γ), WT (but not *Parp1*^-/-^) BM-Mφ exhibited a potent increase in proinflammatory cytokines’ gene expression at 3 h and 18 h (Fig 2A–2C, S3A–S3C Fig, ^*i*^p<0.001).

In agreement with the transcriptional profile, WT BM-Mφ incubated with TEv_Raw_ (± IFN-γ) for 48 h exhibited 76–145%, 50–370%, and 46–80% increase in the release of TNF-α, IL-6, and IL-1β cytokines, respectively (Fig 2D–2F, all, ^+^p<0.001). In comparison, a modest increase in cytokines’ release was observed in *Parp1*^-/-^ BM-Mφ incubated with TEv_Raw_ (± IFN-γ) (Fig 2D–2F). *T*. *cruzi* infection also resulted in a potent increase in proinflammatory cytokines’ release by WT (^*i*^p<0.001), but not in *Parp1*^-/-^, BM-Mφ (Fig 2D–2F).

To validate the *in vitro* findings, we incubated Raw Mφ with plasma TEv of chronically infected WT mice and showed 17.5-fold, 47-fold, and 7.6-fold increase in the secretion of TNF-α, IL-6, and IL-1β cytokines, respectively, as compared to that noted when Mφ were incubated with NEv of non-infected mice (Fig 2G–2I, all, ^+^p<0.001). A slightly higher level of cytokines’ release was observed when Mφ were incubated with TEv_WT_^ch^ + IFN-γ (Fig 2G–2I, all, *p<0.05). Co-incubation with iniparib (selective PARP1 inhibitor) resulted in 80%, 52%, and 43% decline in TEv_WT._^ch^ + IFN-γ-induced TNF-α, IL-6, and IL-1β release, respectively, in Mφ (Fig 2G–2I, all, ^*i*^p<0.01). Similar to the observations with murine TEv, proinflammatory activation of Raw Mφ by TEv_C2C12_ was also arrested by iniparib (S3D–S3F Fig).

Together the results presented in Fig 2 and S3 Fig demonstrate that a) Ev produced during *T*. *cruzi* infection and chronic Chagas disease induce cytokines’ gene expression and synergize with IFN-γ to elicit the proinflammatory cytokines’ release in supernatants of primary and cultured Mφ. Further, b) chemical inhibition or genetic deletion of PARP1 arrested the transcriptional (and translational) activation of proinflammatory cytokine response in Mφ incubated with *T*. *cruzi*-induced Ev. We surmise that PARP1 is an essential transcriptional regulator that transmits the stimulus provided by Ev produced during Chagas disease to signal the proinflammatory cytokines’ expression.

### Compositional analysis of extracellular vesicles produced by *T*. *cruzi* infection

**Fig 3:**
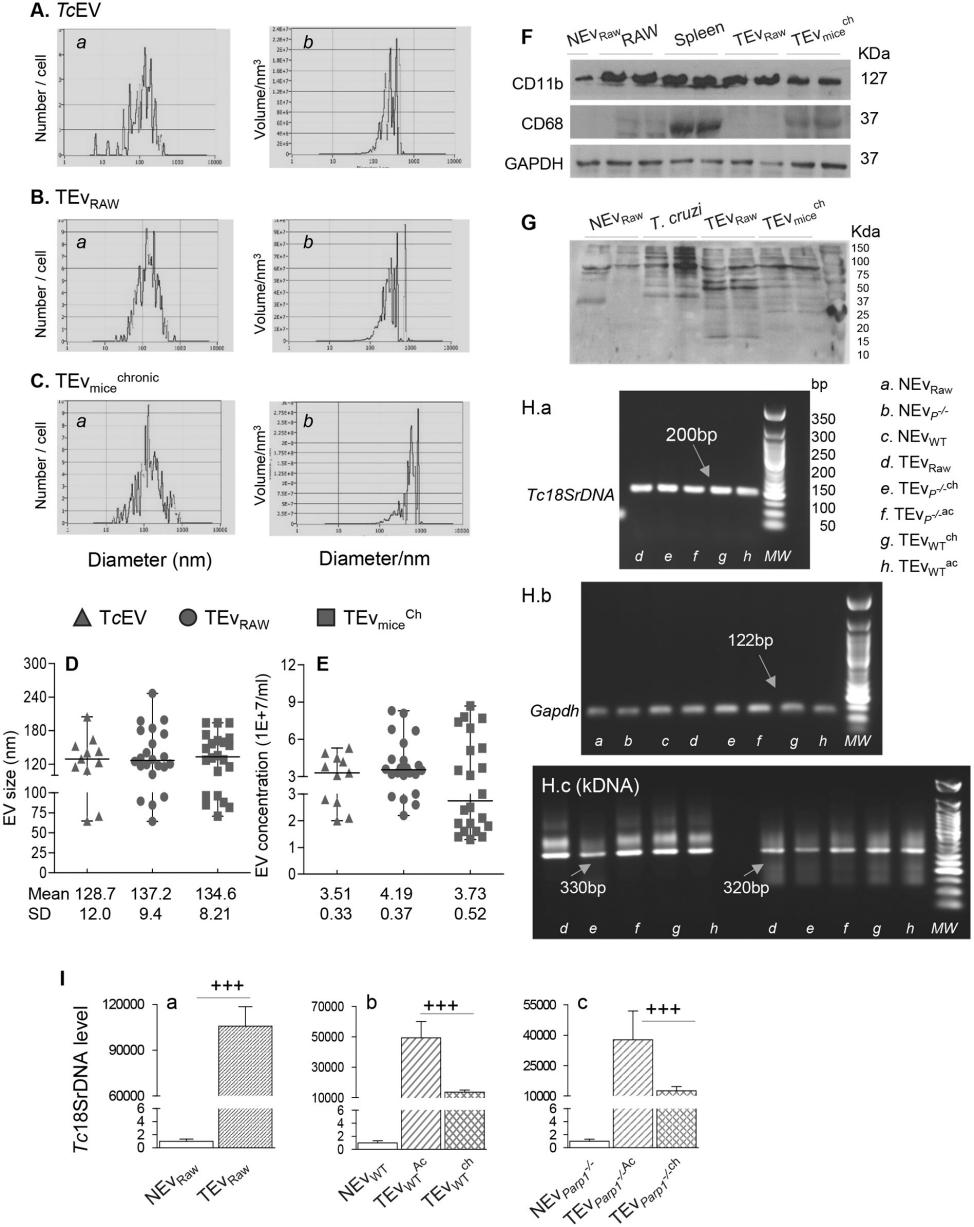
**(A-E) Physical characterization of *Tc*-induced extracellular vesicles**. Shown in ***A-C*** are representative peaks for the absolute number (panels a) and volume per nm^3^ (panels b) in relation to size distribution (diameter/nm) for *Tc*Ev purified from spent media of *T*. *cruzi* culture, and Ev purified from supernatants of infected RAW 264.7 Mφ (TEv_Raw_) and plasma of chronically infected mice (TEv_mice_^ch^). Scatter dot plots for size and concentration measurements of T*c*Ev, TEv_Raw, and_ TEv_mice_^ch^ were obtained from two cycles of observations per sample, scanning 11 cell positions and capturing 60 frames. The mean and the standard deviation values for each measurement is presented below the scattered dot plots ***(D&E)***. **(F&G) Protein markers by western blot analysis**. Purified TEv_Raw_, TEv_mice_^ch^ and controls (NEv_Raw_, and Mφ, splenic, and *T*. *cruzi* lysates, 10 μg) were resolved by SDS-PAGE. Representative western blot images show CD11b, CD68 and, GAPDH levels ***(F)*** and reactivity to anti-*T*.*cruzi* polyclonal sera from infected mice ***(G)***. **(H&I) PCR analysis of DNA in Ev**. Total TEv_DNA_ was purified from TEv isolated from supernatants of infected Raw Mφ and plasma of acutely (ac) and chronically (ch) infected WT and *Parp1*^*-/-*^ mice. Real-time qPCR was performed to amplify *T*. *cruzi-specific 18SrDNA* and murine *Gapdh* sequences. The products of qPCR were resolved by 1.5% agarose gel electrophoresis (***H*, *panel a***). *Tc18SrDNA* levels normalized to *mGapdh* are presented as fold change ± SD (***I*, *panels a-c***) with two biological replicates each and triplicate observations per sample for *panel a* and n = 5 for *panels b & c* (vs. matched control NEv, ^+^p<0.001). The conserved (330 bp) and variable (320 bp) regions of *T*. *cruzi* kinetoplast DNA minicircle were amplified by traditional PCR for 40 cycles and resolved by 1.5% agarose gel electrophoresis *(****I****)*. NEv_DNA_ purified from NEv of non-infected Mφ and mice were used as controls.

We first characterized the physical attributes of TEv by nanoparticle tracking analysis (NTA). Representative images of size and concentration of *Tc*Ev, TEv_RAW_, TEv_WT_^ch^ isolated from *T*. *cruzi* cultures, supernatants of infected Mφ, and plasma of chronically infected WT mice, respectively, are shown in Fig 3A–3C. Dot plots along with mean value ± SD for the size and concentration of Ev in different samples are presented in Fig 3D & 3E. These data showed no major differences in size of the *Tc*Ev released by *T*. *cruzi* as well as *Tc*-induced Ev released by infected cells and mice (Fig 3A–3C panels a & Fig 3D). Ev samples ranged from 70–260 nm in size with mean value of 128.7–137.2 nm and were within the microvesicles size range. Likewise, *Tc*Ev and TEv distribution per mL ranged from 1.5 1E+7–8.7 1E+7 (mean value: 3.51 1E+7–4.19 1E+7), thus suggesting that overall concentration used for NTA were similar for all samples.

Extracellular vesicles harbor nucleic acids and proteins from the cell of their origin and may also uptake other components during transit from the site of their origin to secretory pathway. We performed western blot analysis to examine macrophage markers and parasite protein content in Ev (Fig 3F & 3G). CD11b (Mφ marker) was present in TEv released by infected cells and mice (Fig 3F, top panel) similar to that noted in total lysates of Raw Mφ and splenic cells. Low, but detectable CD11b signal was also noted in NEv_Raw_. CD68 (marker of hematopoietic cells of monocytic lineage) was primarily detected in TEv_mice_^ch^ and splenic lysate (Fig 3F). Probing with polyclonal anti-*T*. *cruzi* sera detected several protein bands in TEv_RAW_ and TEv_mice_^ch^, which were closely matched to those noted in *T*. *cruzi* lysate (Fig 3G). Except for a 75 kDa band, no reactivity of *T*. *cruzi* polyclonal sera was noted with NEv_Raw_.

We examined the DNA content of Ev samples by qPCR and traditional PCR. Representative gel images and average data from > 3 experiments plotted in bar graphs are shown in Fig 3H & 3I and S4 Fig. Real time qPCR showed the presence of murine mitochondrial *Cytb* and *COII* DNA (normalized to *Gapdh*) in all TEv samples as well as in matched NEv controls (S4A–S4F Fig). No significant differences were observed in mtDNA levels in NEv and TEv of different origin. Gel analysis of the qPCR products confirmed the specificity of the amplified bands (S4G–S4I Fig). *T*. *cruzi*-specific *18SrDNA* and *kDNA* bands were detected in TEv isolated from supernatants of infected Raw Mφ and plasma of acutely and chronically infected WT and *Parp1*^-/-^ mice, but were not detected in matched, control NEv isolated from non-infected cells and mice (Fig 3H, panels a and c). *Tc18SrDNA* levels (normalized to *Gapdh*) were maximally noted in TEv_Raw_ and similarly noted in TEv of acutely and chronically infected WT and *Parp1*^-/-^ mice (Fig 3I, panels a-c). Together the results presented in Fig 3 and S4 Fig suggest that a) *Tc*Ev released by *T*. *cruzi* and TEv released by infected cells and mice are of microvesicle size, and b) the TEv carrying Mφ and *T*. *cruzi* proteins and host mtDNA and *T*. *cruzi* DNA are consistently released during the course of CD development. NEv produced by non-infected cells and mice carried similar amounts of host DNA as was noted in TEv, but low amounts of host proteins and no proteins and DNA of *T*. *cruzi* origin were detected in NEv controls.

### Components of TEv that elicit proinflammatory response in Mφ (± PARP1)

**Fig 4:**
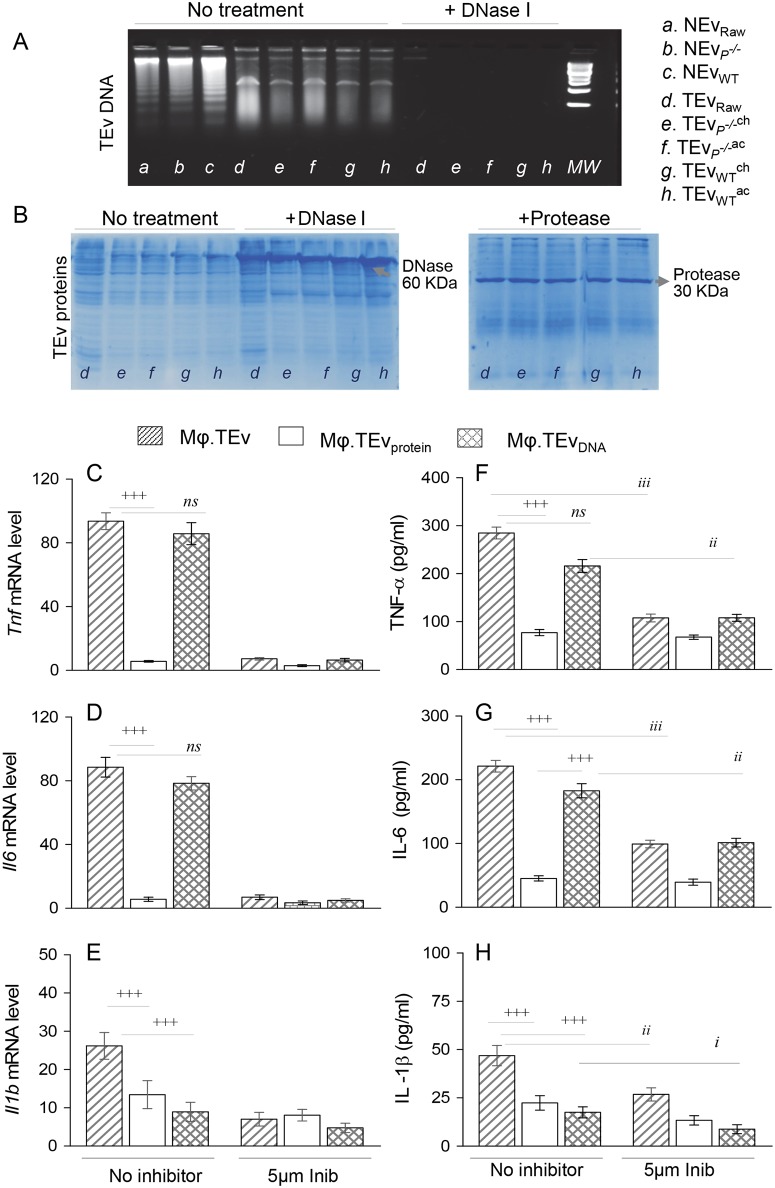
Molecular contents of TEv that induce proinflammatory Mφ activation in PARP1- dependent manner. Supernatants of *T*. *cruzi*-infected Raw 264.7 Mφ were used to isolate TEv, and the latter were processed to purify TEv_DNA_ and TEv_protein_ fractions, as described in Materials and Methods. **(A)** Representative 1.5% agarose gels show the Ev_DNA_ (± DNase I) of TEv of infected Raw Mφ, and acutely and chronically infected WT and *Parp1*^-/-^ mice (controls: Matched NEv). **(B)** Representative 10% PAGE gels stained with Coomassie blue show TEv_protein_ fractions (± DNase I or protease). **(C-H)** Cultured Mφ were incubated with TEv, and DNase-treated TEv_protein_ and protease-treated TEv_DNA_ fractions (± 5 μM iniparib) for 3 h or 48 h. The mRNA levels (3 h) and protein levels (48 h) of TNF-α, IL-6 and IL-1β cytokines were determined by real time RT-qPCR **(C-E)** and ELISA **(F-H)**, respectively. Data in bar graphs are representative of ≥ 2 independent experiments (two biological replicates per treatment and triplicate observations per sample for RT-qPCR analysis; and three biological replicates per treatment and duplicate observations per sample for ELISA), and presented as mean ± SD. Statistical significance is annotated as ^**+**^ (TEv vs. TEv_DNA_ or TEv_protein_ fractions) and ^*i*^ (effect of iniparib on TEv-induced responses); and p values of ≤ 0.05, ≤ 0.01, and ≤ 0.001 are presented by one, two, and three symbol characters, respectively. Horizontal bars denote the compared groups. *ns*: non-significant.

To determine if DNA or proteins carried by TEv stimulate PARP1-mediated cytokine response, we treated TEv with DNase I and protease, and used the treated samples to purify TEv_protein_ and TEv_DNA_ fractions, respectively. We confirmed that DNase I treatment degraded DNA in all Ev samples (Fig 4A) but it did not interfere with protein content of TEv (Fig 4B). Similarly, treatment with protease specifically resulted in significant reduction in protein bands intensity and number in TEv (Fig 4B).

Next, we incubated the cultured Mφ with TEv and TEv_DNA_ and TEv_protein_ fractions (± iniparib), and examined the cytokines’ gene expression by RT-qPCR at 3 h and cytokines’ release by an ELISA at 48 h. Mφ incubated with DNase I-treated, TEv_protein_ fractions exhibited 14–15.5-fold and 13–14.8-fold decline in *Tnf* and *Il6* mRNA levels, respectively (Fig 4C & 4D, all, ^+^p<0.001), and 1.8–2.7-fold and 3.0–3.9-fold decline in TNF-α and IL-6 release, respectively (Fig 4F & 4G, ^+^p<0.001), as compared to that noted in Mφ that were incubated with TEv or protease-treated TEv_DNA_ fractions. Both TEv_DNA_ and TEv_protein_ fractions (vs. TEv) elicited 1.0–1.9-fold lower levels of *Il1b* mRNA and 1.1–1.7-fold lower levels of IL-1β in Mφ (Fig 4E & 4H, ^+^p<0.001). Co-incubation with 5 μM iniparib (selective PARP1 inhibitor) neutralized the TEv- and TEv_DNA_-induced expression of TNF-α and IL-6 cytokines by (Fig 4C, 4D, 4F & 4G, all, ^*i*^p<0.01). Treatment with iniparib also weakened the IL-1β protein levels by 0.8–1.0-fold in Mφ incubated with TEv or TEv_DNA_ (Fig 4H, all, ^*i*^p<0.05). Together these results suggest that DNA (but not protein) contents of TEv produced by *T*. *cruzi* infection provide the major stimulus for the activation of proinflammatory cytokine response in Mφ in a PARP1-dependent manner. NEv isolated from non-infected cells and mice carried similar amounts of host DNA as was noted in TEv (S4 Fig) and these NEv were non-inflammatory (Figs 1–3). These data suggest that parasite DNA, and likely not the host DNA, carried by TEv elicit the Mφ proinflammatory activation. However, these data do not rule out the potential role of epigenetically modified parasite and/or host DNA in signaling proinflammatory response, and this will be determined in future studies.

### Signaling receptors involved in TEv-mediated up regulation of proinflammatory cytokine response in Mφ

**Fig 5:**
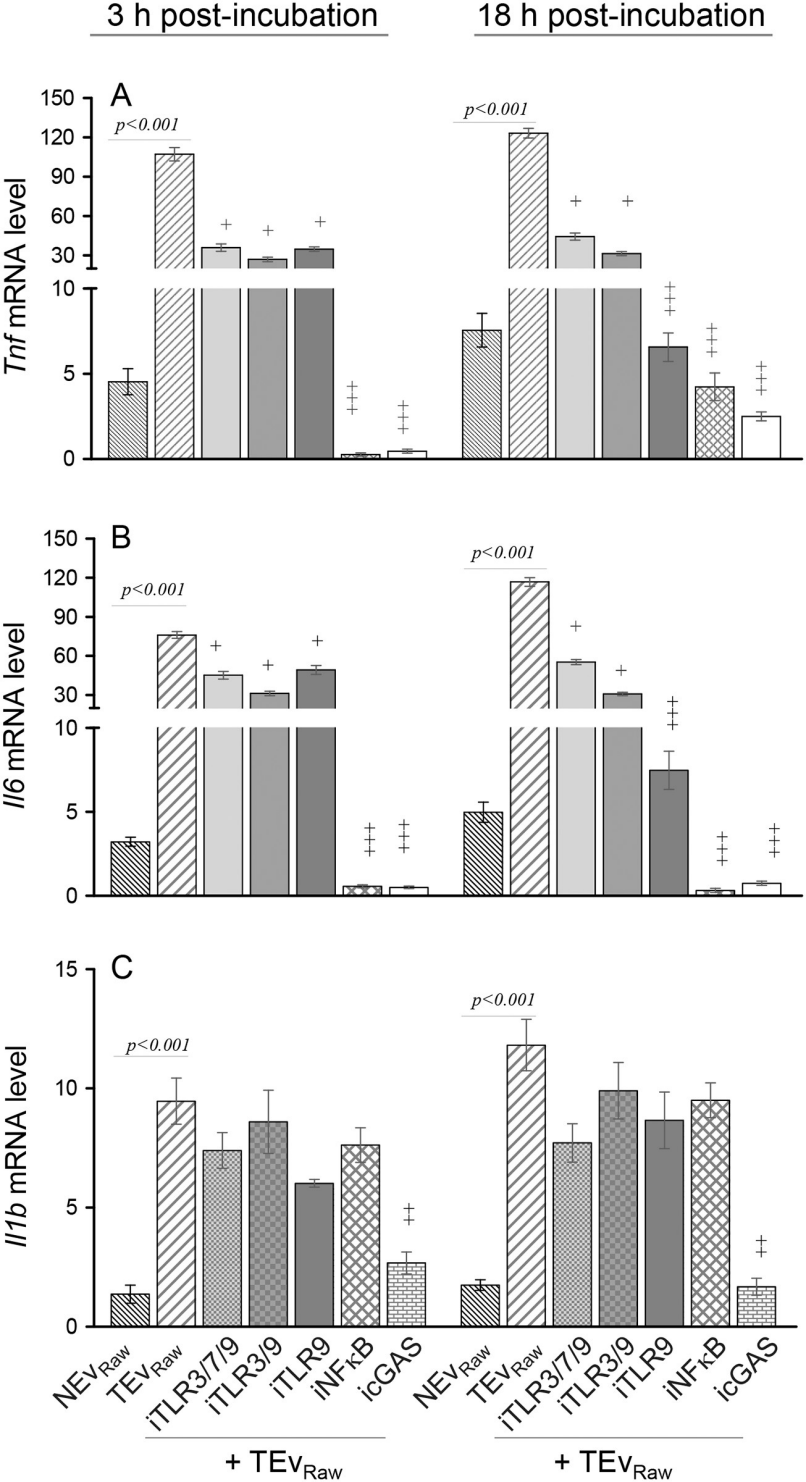
Innate immune receptors engaged in signaling of TEv-induced macrophage activation. NEv and TEv were purified from supernatants of Raw 264.7 Mφ incubated for 72 h with media only or *T*. *cruzi*, respectively. Next, Raw Mφ were incubated with NEv or TEv for 3 h or 18 h in presence or absence of 5 μM chloroquine (inhibits endosomal TLR3/7/9), 5 μM quinacrine (inhibits TLR3/9), 5 μM ODN-2088 (ODN, inhibits TLR9), 10 μM PF-06928215 (inhibits cGAS) or 5 μM JSH-23 (inhibits NF-κB activation). The mRNA levels for genes encoding TNF-α **(A)**, IL-6 **(B)**, and IL-1β **(C)** were evaluated by RT-qPCR. Data are representative of ≥ 2 independent experiments (two biological replicates per treatment and three observations per sample) and presented as mean ± SD. Unless indicated with horizontal bar, statistical significance comparing TEv vs. TEv + inhibitor is annotated as ^**+**^ p ≤ 0.05, ^++^ p ≤ 0.01, and ^+++^ p ≤ 0.001.

Mφ express a variety of pattern recognition receptors (PRR) to recognize pathogen- and damage- associated molecular patterns (PAMPs and DAMPs) to signal the immune activation cascade. Among these, cytoplasmic toll-like receptors TLR3 and TLR7 recognize the single-stranded or double- stranded RNA and TLR9 recognizes DNA and can play a key role in activation of innate immune system. Cyclic GMP-AMP synthase (cGAS) is suggested to recognize genomic DNA damage and trigger innate immune responses through cGMP-mediated activation of STING adaptor protein [16]. To delineate whether TLRs or cGAS recognize *T*. *cruzi*-induced Ev to signal the downstream cascade for cytokines gene expression, we incubated the cultured Mφ with TEv_Raw_ in presence of specific inhibitors of TLR3/7/9, cGAS, and NF-κB transcription factor for 3 h or 18 h, and monitored the cytokines’ gene expression by RTqPCR. As expected from Fig 4, incubation of Mφ with TEv_Raw_ elicited a potent increase in cytokines’ gene expression at 3 h and 18 h (vs. NEv control, all, p<0.001). Co-incubation with chloroquine (inhibits endosomal TLR3/7/9), quinacrine (inhibits TLR3/9) and ODN-2088 (specific inhibitor of TLR9) decreased the TEv-induced expression of *Tnf* and *Il6* by 35–75% at 3 h and 52–95% at 18 h post-incubation (Fig 5A & 5B). Among these, ODN-2088 inhibitor of TLR9 was most effective in suppressing the *Tnf* and *Il6* expression in TEv-stimulated Mφ (Fig 5A & 5B, all, ^+^p<0.01). In comparison, short-term treatment with cGAS antagonist (PF-06928215) was sufficient to cause a potent decline in TEv-induced cytokines’ expression in Mφ. This was evidenced by 230-fold, 148-fold, and 2.5-fold decline in *Tnf*, *Il6*, and *Il1b* mRNA levels, respectively, at 3 h post-incubation (Fig 5A–5C, all, ^+^p<0.001) that was not further changed at 18 h (Fig 5A–5C). In presence of 5 μM JSH-23 (inhibits NF-κB transcriptional activity), the expression of *Tnf* and *Il6* was completely abolished in TEv-stimulated Mφ at 3 h and 18 h post-incubation (Fig 5A & 5B, all, ^+^p<0.001). NF-κB inhibition did not alter the TEv-induced *Il1b* gene expression in Mφ (Fig 5C). Together, these results suggest that a) TLR9 and cGAS signal cytokines gene expression (*Tnf* and *Il6* >> *Il1b*) through NF-κB activation in TEv-treated Mφ. The pronounced inhibition of cytokines’ expression by PF-06928215 (vs. ODN-2088) at 3 h suggests that cGAS-STING (and not TLR9-MyD88) pathway is the early responder in recognizing TEv stimulus and signaling Mφ proinflammatory cytokine gene expression.

### Synergistic role of cGAS and PARP1 in signaling NF-κB activity in Mφ (± TEv)

**Fig 6:**
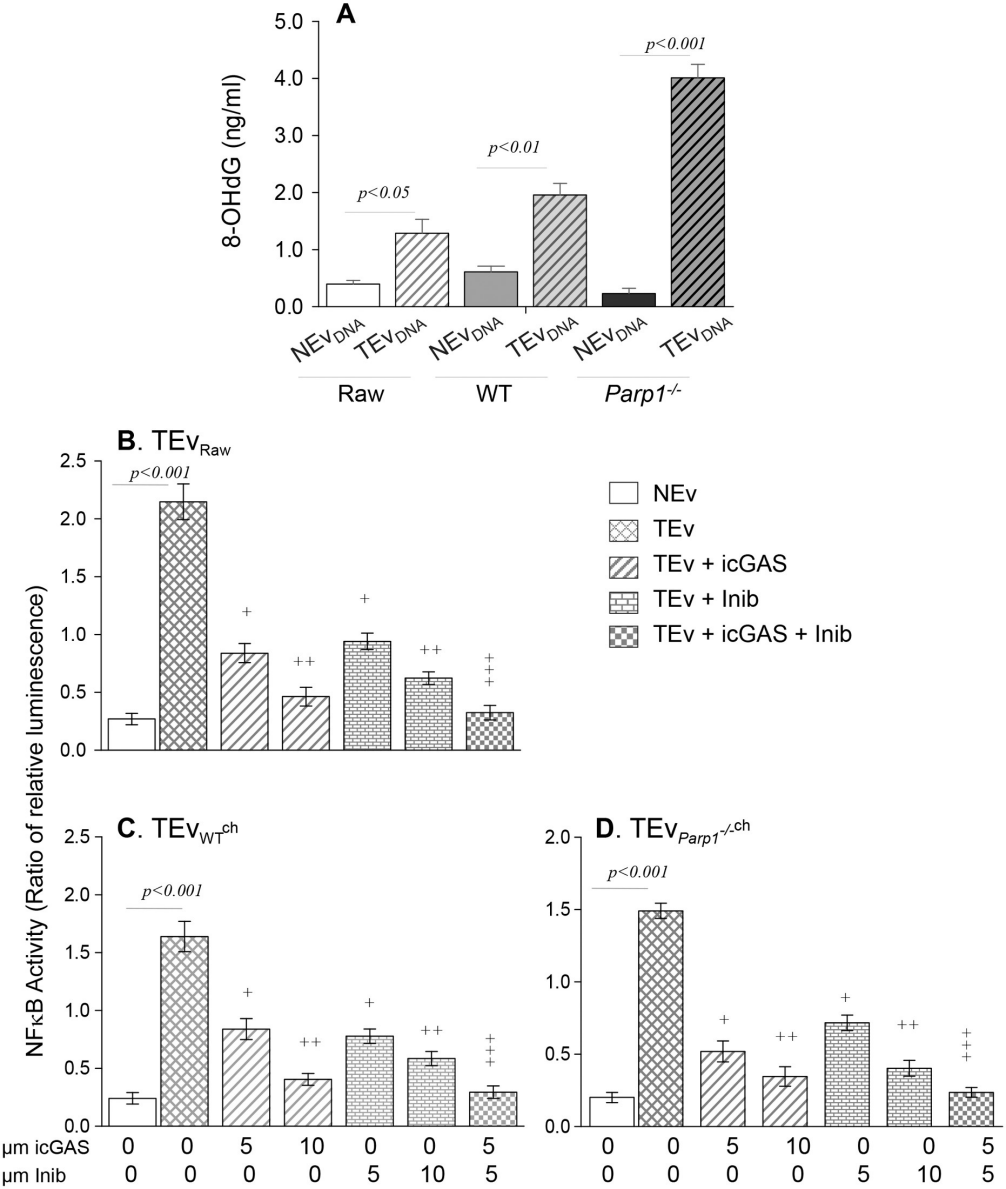
**(A) *T*. *cruzi*-induced Ev**_**DNA**_ **is oxidized**. NEv and TEv were isolated from normal and *T*. *cruzi*-infected Raw Mφ (triplicate samples per group), and from normal and chronically infected WT and *Parp1*^-/-^ mice (n = 10 mice per group). Total DNA was purified from all Ev preparations, and an OxiSelect DNA Damage ELISA was performed to evaluate the 8-hydroxy-2’- deoxy guanosine (8-OHdG) levels. **(B-D) Cross talk between cGAS and PARP1 regulates the TEv-induced NF-κB activity in Mφ**. Raw 264.7 Mφ were transfected with NF-κB luciferase reporter and control renilla luciferase. Transfected cells were incubated for 3 h with TEv_RAW_ purified from supernatants of *T*. *cruzi*-infected Mφ **(B)**, or TEv purified from plasma of chronically infected WT **(C)** and *Parp1*^-/-^ **(D)** mice (**±** 5 μM or 10 μM concentrations of PF-06928215 (inhibits cGAS) and iniparib (inhibits PARP1)). Cells were used in a Dual Luciferase (Firefly-Renilla) Assay, and the ratio of relative luminescence for firefly to renilla luciferase (control) was calculated as a measure of NF-κB activity. Matched NEv were used as controls. Data are representative of ≥ 2 independent experiments (six biological replicates per treatment) and presented as mean ± SD. The p values of ≤ 0.05, ≤ 0.01, and ≤ 0.001 are presented by +, ++, and +++ symbol characters, respectively (TEv vs. TEv + inhibitor).

Since both cGAS and PARP1 are activated by DNA damage, we first evaluated the oxidized DNA content in Ev produced by *T*. *cruzi* infection of cells and mice. TEv released in supernatants of *T*. *cruzi-*infected Mφ and in plasma of chronically infected WT and *Parp1*^-/-^ mice exhibited 2.2-fold, 2.25- fold, and 16.4-fold increase in the 8-OHdG contents, respectively, in comparison to matched NEv controls (Fig 6A, all, p<0.05). The Ev of chronically infected *Parp1*^-/-^ mice carried the most amount of oxidized DNA. These results showed that *T*. *cruzi* infection and chronic Chagas disease produce DNA oxidative damage and DNA fragmentation, and PARP1 knockdown increases the release of oxidized DNA encapsulated in secreted TEv.

Then, we performed a dual luciferase reporter assay to determine if cGAS and PARP1 independently or synergistically signal NF-κB activation in Mφ. For this, Raw Mφ were transiently transfected with NFκB-Luc reporter plasmid and pREP7-Rluc (transfection efficiency control), and then incubated for 3 h with TEv isolated from supernatants of *T*. *cruzi-*infected cells, or from plasma of chronically infected WT and *Parp1*^-/-^ mice. Mφ were incubated with Ev in presence of 5 μM and 10 μM concentrations of PF-06928215 (cGAS inhibitor) and/or iniparib (PARP1 inhibitor) to understand the role of these DNA sensing molecules in signaling NFκB transcriptional activation. Mφ incubated with TEv_Raw_ (vs. NEv_Raw_) exhibited 6.7-fold increase in NF-κB-luciferase activity (normalized to *Renilla* luciferase, p<0.001) that was inhibited by 65–80% and 60–75% in presence of cGAS inhibitor and PARP1 inhibitor, respectively (Fig 6B, ^+^p<0.05–0.01). In comparison, co-treatment with cGAS and PARP1 inhibitors (5 μM each) resulted in >85% inhibition of NF-κB activity in TEv_Raw_-stimulated Mφ (Fig 6B, ^+^p<0.001). Likewise, TEv of chronically infected WT mice elicited 6-fold increase in NF-κB-luciferase activity (vs. NEv of uninfected mice, p<0.001), and TEv_WT_^ch^-induced NFκB activity was inhibited by 53–75% and 55–67%, respectively, in presence of cGAS and PARP1 inhibitors (Fig 6C, all, ^+^p<0.05–0.01); and by >80% when TEv_WT_^ch^-stimulated Mφ were treated with both inhibitors (5 μM each, Fig 6C, ^+^p<0.001). Unexpectedly, TEv isolated from peripheral blood of chronically infected *Parp1*^-/-^ mice were also proinflammatory, evidenced by 6.5-fold increase in NF-κB-luciferase activity in comparison to that noted in Mφ incubated with NEv of *Parp1*^-/-^ mice (Fig 6D, p<0.001). As above, we noted 65–75% and 55–75% decline in TEv_*Parp1*_^*-/-*ch^-induced NF-κB activity in presence of cGAS and PARP1 inhibitors, respectively (Fig 6D, ^+^p<0.05–0.01), while co-treatment with cGAS and PARP1 inhibitors (5 μM each) resulted in >80% inhibition of TEv_*Parp1*_^*-/-*ch^-induced NF-κB activity in Mφ (Fig 6D, p<0.001). Together, these results suggest that a) TEv released in response to *T*. *cruzi* infection and chronic disease carry oxidized DNA, and b) Mφ uptake of TEv carrying oxDNA is sensed by cellular DNA response element cGAS to signal NF-κB transcriptional activation. The findings that TEv_*Parp1*_^*-/-*ch^ carry similar amount of *Tc*DNA and murine mtDNA as was noted in TEv_WT_^ch^ (Fig 3H & 3I), and TEv_*Parp1*_^*-/-*ch^ also signaled NFκB activity in Mφ, while PARP1 inhibitor prevented the TEv_WT_ch—induced NF-κB activity, we surmise that c) PARP1 is not required for the generation of TEv of proinflammatory phenotype in CD. Instead, d) PARP1 synergizes with cGAS in signaling the NF-κB transcriptional activity in TEv stimulated Mφ.

### Tissue inflammatory infiltrate in WT and *Parp1*^-/-^ mice

**Fig 7:**
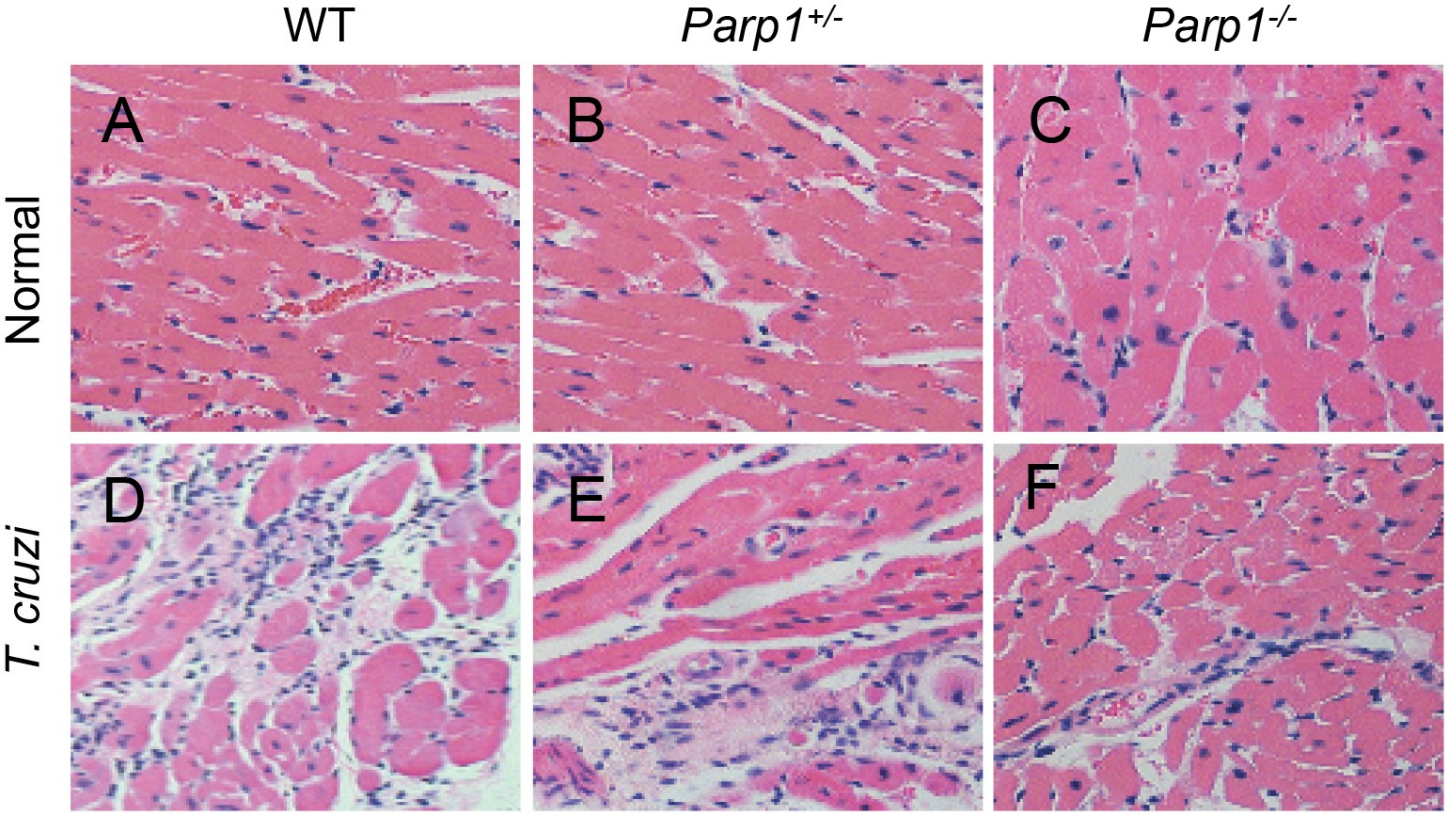
Histological evaluation of myocardial inflammation in chronically infected mice (± PARP1). Mice (WT, *Parp1*^+/-^, and *Parp1*^-/-^) were challenged with *T*. *cruzi* (10,000 parasites per mouse) and euthanized at 150 days post-infection. Paraffin-embedded left ventricular heart tissue sections (5 μM) were examined by hematoxylin/eosin staining (blue: nuclear; pink: muscle/cytoplasm). Shown are representative H&E stained images of tissue sections from **(A-C)** non-infected and **(D-F)** chronically infected WT ***(A&B)***, *Parp1*^*+/-*^ ***(B&E)***, and *Parp1*^*-/-*^ ***(C&F)*** mice. Average histological score ± SD values were derived from analysis of n = 3 mice per group, 2–3 tissue sections per mouse, 10 microscopic fields per tissue section, and discussed in Results section.

Macrophage uptake of inflammatory TEv produced during chronic infection can sustain persistent inflammation, a key cause for left ventricular dysfunction in Chagas disease. We, therefore, first determined if inhibition of PARP1 would arrest chronic inflammation in Chagas disease. Histological evaluations showed the extent of inflammatory infiltrate in heart tissue of chronically infected WT mice (Fig 7D, score: 4.5 ± 0.22, p < 0.05) was significantly increased as compared to the non-infected WT mice (Fig 7A, score: 0.30 ± 0.06). Extensive inflammatory foci as well as diffused inflammation, interstitial edema, and loss of tissue integrity were visible in all tissue sections of CD WT mice. In comparison, myocardial persistence of inflammatory infiltrate was significantly decreased in chronically infected *Parp1*^+/-^ (Fig 7E, score: 2.5 ± 0.19, p < 0.05) and *Parp1*^-/-^ (Fig 7F, score: 1.9 ± 0.27, p < 0.05) mice.

**Fig 8:**
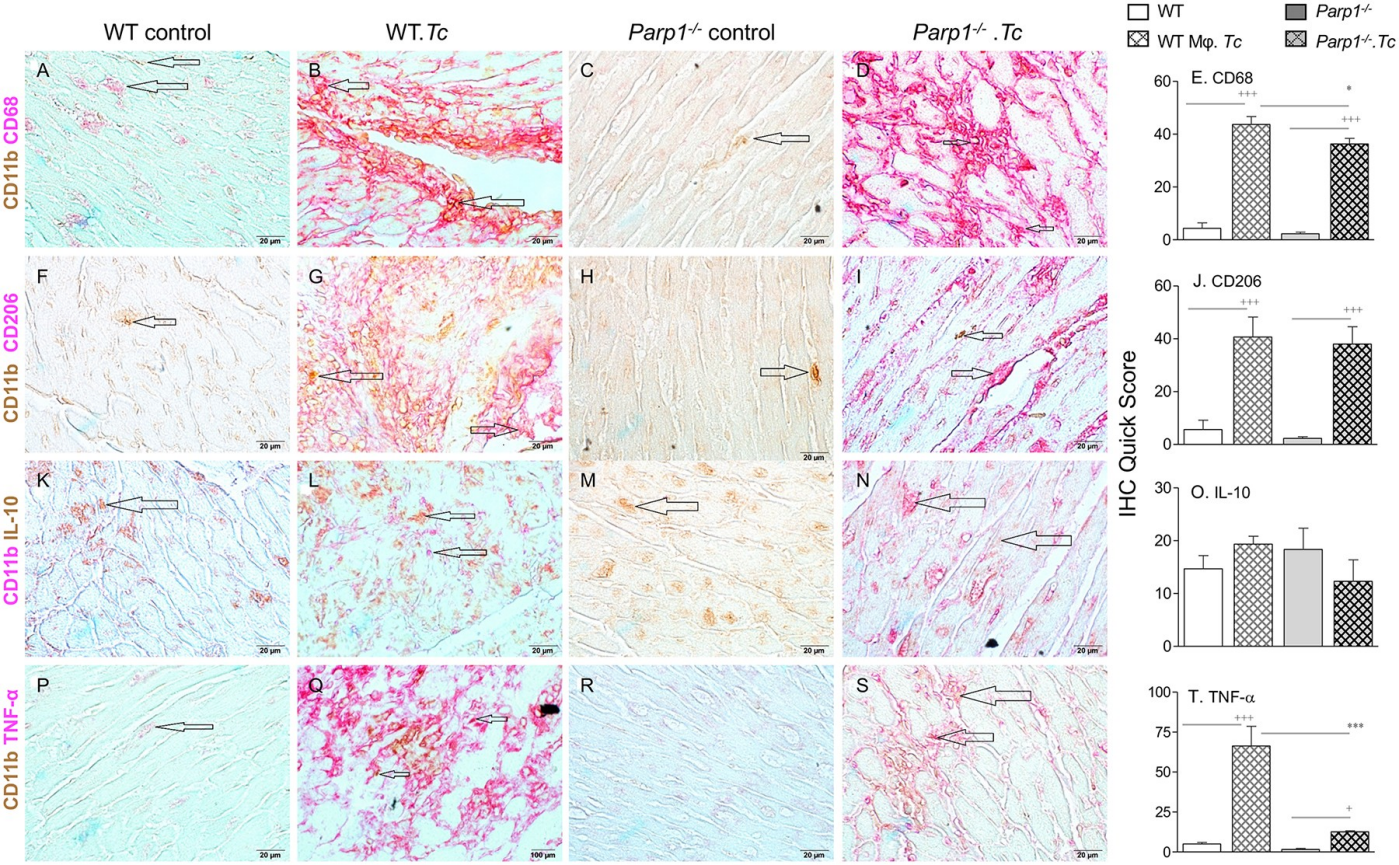
Myocardial macrophage profile in Chagas disease (± PARP1). Mice (WT and *Parp1*^-/-^) were euthanized at 150 days post-infection and myocardial tissue sections of non-infected and infected mice were subjected to immunohistochemistry staining. Shown are representative cardiac tissue images of WT and *Parp1*^-/-^ mice (± *T*. *cruzi* infection, 60X magnification). Bar graphs show semi-quantitative, immunohistochemistry quick score ± SD (n = 3 mice per group, two tissue sections per mouse, 9 microscopic fields per tissue section, 20X magnification), and significance is annotated as ^+^ infected vs. non-infected and *WT.*T*. *cruzi* vs. *Parp1*^-/-^.*T*. *cruzi*. The p values of ≤ 0.05, ≤ 0.01, and ≤ 0.001 are presented by one, two, and three symbol characters, respectively. **A-E**: CD11b^+^CD68^+^ (Mφ markers), **F-J**: CD11b^+^CD206^+^ (detects Mφ of anti-inflammatory phenotype), **K-O**: CD11b^+^IL-10^+^ (indicates immuno-regulatory activation of Mφ), and **P-T**: CD11b^+^TNF-α^+^ (indicates proinflammatory activation of Mφ).

Finally, immunohistochemical staining of tissues was done to assess the effect of PARP1 depletion on myocardial Mφ activation status in Chagas disease. These data showed that the myocardial infiltration of CD11b^+^ and CD11b^+^CD68^+^ cells was significantly and equally increased in chronically infected WT and *Parp1*^-/-^ mice (vs. matched controls, Fig 8A–8E, S5 Fig, all, ^+^p<0.001). Likewise, CD11b^+^CD206^+^ staining (indicates immunoregulatory phenotype) was increased by > 6-fold in the heart tissue of chronically infected WT and *Parp1*^-/-^ (vs. matched non-infected) mice (Fig 8F–8J, ^+^p<0.001). We observed no statistically significant changes in the overall myocardial staining for CD11b^+^IL-10^+^ cells among any of the groups (indicates immunoregulatory function, Fig 8K–8O). However, CD11b^+^TNF-α^+^ staining (indicates proinflammatory functional profile) was increased by 12-fold in the myocardium of chronically infected (vs. non-infected) WT mice (Fig 8P, 8Q & 8T, ^+^p<0.001). In comparison, chronically infected *Parp1*^-/-^ mice exhibited 4.2-fold decline in myocardial CD11b^+^TNF-α^+^ staining (compare Fig 8S & 8Q, *p<0.001). Together, the results presented in Figs 7 and 8 suggest that PARP1 does not regulate the myocardial frequency of Mφ or immunoregulatory response, and instead PARP1 contributed to proinflammatory phenotypic/functional profile of tissue Mφ in Chagas disease. PARP1 depletion was beneficial in arresting the myocardial inflammatory infiltrate through dampening the proinflammatory activation of tissue Mφ in Chagas disease.

## Discussion

Early studies have shown that parasite proteins are transferred from infected muscle, neuronal, epithelial, and fibroblast cells to uninfected host cells, though this antigen transfer was not observed in lymphocytes and erythrocytes [17]. Recent literature documented that this jumping of *T*. *cruzi* antigens occurs through the release of membrane vesicles (also called extracellular vesicles). It was shown that *T*. *cruzi* sheds compositionally different Ev depending on the developmental stage and virulence of the parasite strain [18, 19], Ev shed by infective trypomastigote form of the parasite have high fusogenic potential with the host cell membranes [20], and contact with infective forms of the parasite also stimulated Ca^2+^-dependent shedding of membrane vesicles from THP-1 Mφ [21]. These and other studies did not explore the signaling cascades by which *T*. *cruzi* stimulates formation of membrane vesicles within itself or on the host cell membranes, though it was proposed that the host and parasite Ev may maintain cellular activation in CD [7]. Indeed, we recently showed that Ev released by human PBMCs exposed to *T*. *cruzi* infection activated a proinflammatory gene expression profile in THP-1 Mφ [3], a finding that strongly suggested that *T*. *cruzi* influences the host cell juxtacrine/paracrine Ev release and impacts the surrounding infected/non-infected cells and tissues. Herein, we provide the first evidence that Ev are released from immune and non-immune cells during *T*. *cruzi* infection and chronic CD progression, and these *T*. *cruzi*-induced Ev (TEv) shape the inflammatory state in chronic CD. Our findings are relevant to transmission of inflammatory signals in Chagas disease.

Seropositive individuals are categorized as indeterminate (C0) when they exhibit no symptoms of heart involvement, and progress through C1-C3 stages of clinical Chagas disease presented with increasing severity of structural and functional alterations in the heart [22, 23]. While the indeterminate clinical form is biased towards an anti-inflammatory profile, the C1-C3 CD patients routinely present proinflammatory profile [6, 28–30] [6, 24–26] associated with a systemic increase in TNF-α^+^ monocytes [27–29], oxidative stress (e.g. lipid hydroperoxides) [30, 31], and an abundance of CD8^+^T cells that express inflammatory cytokines and cytotoxic molecules [32]. Microscopic examination of tissues also routinely shows that *T*. *cruzi* infection causes extensive myocardial damage, characterized by vacuolization, myocytolysis, and myofibrillar degeneration and these changes are invariably associated with intense infiltration of macrophages followed by lymphocytes during acute infection. While parasite burden is controlled, tissue mononuclear cells remain active during chronic CD (reviewed in [2]). Our findings of *T*. *cruzi* DNA and proteins in TEv, and TEv stimulation of proinflammatory Mφ that are also excellent antigen presenting cells (APC) suggest that *T*. *cruzi* antigens carried by TEv might also shape the APC-T cell dependent response in patients with different forms of CD. If this is proven in future studies, it will solve the decades old question of how the stimulus is provided for chronic inflammatory state in CD and provide important information regarding the TEv’s association with clinical disease progression.

While Mφ are the major innate immune cells that exert trypanocidal effects by producing ROS and NO, we have found that non-immune cells (skeletal muscle, cardiomyocytes) also respond to *T*. *cruzi* by increased release of ROS of mitochondrial origin [14]. The ROS/NO exert cytotoxic effects through oxidation of cellular components including DNA, proteins, and lipids and are not discriminatory of the parasite and the host cells [33]. Indeed, we have shown that 8-hydroxy-2’-deoxy guanosine (8-OHdG, marker of DNA oxidative damage) was enhanced in *T*. *cruzi*-infected cardiomyocytes [14] and myocardium of chronically infected Chagas mice and patients [34]. In this study, we demonstrate that these damaged DNA fragments are encapsulated in Ev (Fig 6) and provide stimulus for proinflammatory activation of macrophages (Fig 4). Indeed, Ev of different organisms have been described as PAMPs and promoters of the innate and adaptive immune responses [35]. The Ev shed by axenic cultures of *T*. *cruzi* were enriched in glycoproteins of the gp85/trans-sialidase (TS) superfamily and other α-galactosyl (α-Gal)-containing glycoconjugates, and stimulated TLR2, proinflammatory cytokines (TNF-α and IL-6), and NO in Mφ [18]. Our data provide first evidence that Ev produced during *T*. *cruzi* infection and chronic Chagas disease are DAMPs that promote NF-κB-mediated proinflammatory cytokines’ production through the engagement of DNA-sensing innate immune receptors (Fig 5). While TLR9 is usually activated by unmethylated CpG sequences in ssDNA molecules, cGAS has emerged as a major sensor of genomic dsDNA damage and it elicits innate immune responses through cGMP-mediated activation of STING adaptor protein [16]. Our finding of an early and potent role of cGAS (than TLR9) in eliciting TEv_DNA_-dependent Mφ activation allows us to propose that genomic DNA damage of parasite and host cells (instead of CpG DNA content in the genomic DNA of the parasite or host cells) serve as the primary stimulus in engaging DNA sensing innate immune receptors and Mφ activation in the context of Chagas disease progression (Fig 5).

The catalytic activity of PARP1 promotes post-translational modification of self and a range of other proteins, and it is believed to be crucial for mediating multiple DNA damage repair pathways. PARP1 is also expressed by *T*. *cruzi* [36]; and PARP1 expression was increased in human cardiomyocytes [14] and in the myocardium of mice infected by *T*. *cruzi* [15]. PARP1 chemical inhibition or genetic deletion preserved the left ventricular function that otherwise was compromised in Chagas WT mice [15]. Our findings in the present study show that *Parp1* deletion was beneficial in controlling the myocardial inflammatory infiltrate, especially the TNF-α-expressing Mφ, in Chagas disease (Figs 7 and 8). These studies imply that PARP1 contributes to Chagas cardiomyopathy through its effects on cardiomyocytes and Mφ. In cardiomyocytes, PARP1 was activated in response to *T*. *cruzi* induced mtROS/DNA damage, and PARP1 facilitated the assembly of the NF-κB transcription complex and cytokine gene expression through post-translational modification of RelA (p65)- interacting nuclear proteins [14]. In this study, we provide evidence that Ev_oxDNA_ released in supernatants of infected cells and in plasma of chronically infected mice stimulate PARP1 activation in Mφ. Further, Mφ-PARP1 complemented the cGAS in stimulating the NF-κB-dependent cytokine gene expression in response to Ev_oxDNA_ produced during *T*. *cruzi* infection. In this context, PARP1 likely served as a cytoplasmic sensor along with cGAS to activate innate signaling cascade (Fig 6). A recent study showed that while phosphorylation of cGAS at Tyr215 (by B-lymphoid tyrosine kinase) facilitates its cytosolic retention, DNA damage induced nuclear translocation of cGAS occurs in importin-α-dependent manner, and in the nucleus cGAS interacted with PARP1 and impeded the formation of the PARP1-Timeless complex and suppressed the homologous-recombination-mediated DNA repair [37]. Whether cGAS directly (or indirectly) interacts with PARP1 in cytosolic and/or nuclear fraction to stimulate Ev_oxDNA_-dependent, NF-κB-mediated proinflammatory response in CD remains to be seen in future studies. However, we surmise that PARP1 is a potential target for controlling chronic inflammatory pathology and Chagas cardiomyopathy. Our proposal is supported by the findings that chemical inhibition or genetic deletion of PARP1 significantly decreased the myocardial inflammatory infiltrate (specifically the macrophages of proinflammatory phenotype) and improved the left ventricular function in Chagas mice.

In summary, we have shown that *Tc*Ev released by *T*. *cruzi* and TEv released by host during *T*. *cruzi* infection and disease progression shape the activation of Mφ that, in turn, augment the chronic proinflammatory state in Chagas disease. Damaged DNA fragments encapsulated within *T*. *cruzi*-induced Ev, are the key biocomponent that promote NF-κB-mediated proinflammatory cytokine production in Mφ through the engagement of cytosolic DNA sensors cGAS and PARP1. Whether TEv also provide antigenic stimulus and PARP1-cGAS induce antigen presenting capacity of the Mφ and support chronic activation of cytotoxic CD8^+^ T cells that are known to be pathologic in human Chagas disease remains to be determined in future studies. We propose that small molecule PARP1 inhibitors offer a potential therapy for controlling the pathologic chronic inflammation in Chagas disease through modulation of the Mφ signaling of cGAS- NF-κB pathway.

## Materials and methods

### Ethics statement

All animal experiments were performed by following the NIH guidelines for Care and Use of Experimental Animals, and in accordance with protocols approved by the Institutional Animal Care and Use Committee at the University of Texas Medical Branch, Galveston (protocol number: 0805029).

### Mice, cell culture, and parasites

B6129S/J (Wild type [WT]) and 129S-*Parp1*^*tm1Zqw*^/J (*Parp1*^*-/-*^) mice were purchased from Jackson Laboratory (Bar Harbor, ME). The *Parp1* gene deletion in *Parp1-/-*mice was confirmed by genotyping for *Parp1* by a standard PCR and by examining *Parp1* mRNA expression by RT-qPCR [15].

For cell cultures, fetal bovine serum (FBS, Invitrogen, Carlsbad, CA) was heat inactivated at 56°C for 30 minutes with intermittent shaking before use in medium. Murine bone marrow (BM) cells were isolated from the femurs of mice by following a standard protocol, and either used immediately or stored at -80°C in 80% heat-inactivated FBS (ha-FBS) with 20% DMSO. The BM cells were suspended in RPMI medium containing 10% ha-FBS, 2-mmol/L glutamine, 100 IU/mL penicillin, 100-μg/mL streptomycin (Corning, Corning, NY), added to 6-well plates (5 x 10^6^ cells per ml per well), and incubated at 37°C in 5% CO_2_ in presence of 20 ng/mL of macrophage colony stimulating factor (M-CSF; Millipore, Burlington MA) [38]. The culture medium and M-CSF were replenished every two days, and cells were incubated for nine days allowing the monocyte progenitor cells to mature as Mφ. Raw 264.7 murine Mφ (ATCC TIB-71) were cultured in complete high glucose Dulbecco’s modified Eagle’s medium (DMEM) containing 10% ha-FBS. The C2C12 mouse myoblast cells (ATCCC CRL-1772) were cultured in complete RPMI 1640 medium containing 5% or 10% ha-FBS.

*T*. *cruzi* (SylvioX10/4, ATCC 50823) trypomastigotes were propagated by *in vitro* passage in C2C12 cells. All chemicals used in the study were of molecular grade and purchased from Sigma-Aldrich unless otherwise specified. Protein levels in the samples were determined by using the Bradford Protein Assay (Bio-Rad, Hercules, CA).

### Generation, isolation, and fractionation of extracellular vesicles (Ev)

The differential centrifugation protocol for the enrichment of Ev consisting microvesicles (100–1000 nm) and apoptotic bodies (1000–5000 nm) is described previously [39]. Briefly, *T cruzi* trypomastigotes (1X 10^7^/10 mL) were incubated in serum free RPMI media for 72 h at 37°C at 5% CO_2_. Cells (C2C12 or Raw 264.7) were seeded in T25 or T75 cell culture flasks, and at 70% confluency, infected with *T*. *cruzi* trypomastigotes (cell: parasite ratio: 1:3). Cells were incubated at 37°C at 5% CO_2_ for 24 h, 48 h, or 72 h in serum free RPMI or DMEM medium. The culture supernatants were centrifuged at 4000 g for 10 minutes to pellet the cell debris and parasites. Then culture supernatants were subjected to three series of centrifugation at 4°C for 30 min each at 20,000 g. The pelleted Ev samples from each centrifugation were washed, resuspended at 10-fold concentration in serum free RPMI medium, and stored at -80°C.

Mice (WT and *Parp1*^*-/-*^, 6-weeks old, n = 10 per group) were infected with *T*. *cruzi* trypomastigotes (10,000/mouse), and euthanized at 30 days and 150 days post-infection (pi) corresponding to acute parasitemia and chronic disease phase, respectively [40, 41]. The EDTA blood samples were centrifuged for 20 minutes at 2000 g to separate plasma. Plasma samples were centrifuged at 4°C for 30 min at 20,000 g, and the pelleted Ev samples were washed and stored at 10-fold concentration in serum free RPMI medium, as above.

For fractionation, Ev samples were treated with DNase I (1 U/μL, EN0521, Thermo Fisher Scientific) at 37°C for 30 min to degrade Ev-associated membrane bound contaminant DNA followed by inactivation of DNase with EDTA. Then Ev samples were treated with 0.5% Triton X-100 in 1X PBS for 10 min at 25°C. The permeabilized Ev samples were incubated for 5 min each with protease cocktail (0.03 U/g, P-311, Sigma-Aldrich) to degrade proteins and 1 mM phenylmethanesulfonyl fluoride (PMSF) to inactivate the proteases, and Ev_DNA_ was extracted by using DNeasy blood and tissue Kit (Catalog; 69504, Qiagen, Hilden, Germany). In other studies, permeabilized Ev samples were sequentially treated with DNase I (1 U/μL, EN0521, Thermo Fisher Scientific) at 37°C for 30 min to degrade Ev_DNA_ and 50 mM EDTA for 10 min to inactivate DNase I and used as a source of Ev_protein_ fraction. In all cases, Ev and Ev fractions were stored at -80°C at 10-fold concentration of the original volume and used at 1:10 ratio (Ev: culture medium, v/v) to obtain biological levels.

### Treatment of Mφ with Ev

Raw 264.7 Mφ and murine BM-derived Mφ (WT and *Parp1*^-/-^) were seeded in 24-well (5×10^4^ cells/300 μL/well) or 6-well (5x10^6^ cells/mL/well) plates and incubated for 2 h to allow the cells to adhere. Macrophages were incubated in triplicate in serum free medium with *Tc*Ev isolated from *T*. *cruzi* axenic cultures, TEv isolated from culture supernatants of *T*. *cruzi*-infected cells or from plasma of infected mice (10% media/plasma equivalent), or TEv fractions. NEv isolated from media alone, supernatants of non-infected cells or plasma of non-infected mice were used as matched controls. Macrophages (± Ev or Ev fractions) were incubated for 3, 6, 18, or 48 h in the presence and absence of 20 ng/mL IFN-γ (11276905001, Sigma-Aldrich) or 5–10 μM iniparib (selective PARP1 inhibitor, S1087, Selleck Chemicals, Houston TX). For signaling studies, Raw 264.7 Mφ were incubated with TEv or NEv in the presence or absence of 5 μM chloroquine (inhibits endosomal TLRs, tlrl-chq, Invivogen, San Diego, CA), 5 μM quinacrine (inhibits TLR3/TLR9, NBP2-29385, Novus biological, Littleton, CO), 5 μM ODN-2088 (TLR9 antagonist, tlrl-2088, Invivogen), 5–10 μM PF-06928215 (cGAS inhibitor, PZ038, Sigma-Aldrich) or 5 μM JSH-23 (NFκB inhibitor, 481408-M, Millipore). In all experiments, cells and supernatants were stored at -80°C. Simultaneously, Raw 264.7 Mφ or C2C12 cells incubated with and without *T*. *cruzi* (cell to parasite ratio, 1:3) in complete DMEM or RPMI medium at 37°C/5% CO_2_ for 48 h were used as positive and negative controls, respectively.

### Cytokine levels by an enzyme-linked immunosorbent assay (ELISA)

Culture supernatants were collected from macrophages (Raw 264.7 or murine bone marrow-derived) or C2C12 cells incubated with NEv, TEv, Ev fractions, or *T*. *cruzi* (± IFN-γ and/or various inhibitors) as described above. Culture supernatants were then utilized for the measurement of cytokines’ release (IL-1β, IL-6, and TNF-α) by using murine cytokine ELISA kits (eBiosciences, San Diego, CA) according to the manufacturer’s specifications. Standard curves were prepared by using recombinant cytokines (4 pg/mL– 10 ng/mL).

### Real time RT-qPCR

Macrophages (± Ev, Ev fractions, *T*. *cruzi*, or inhibitors) were snap-frozen in liquid nitrogen and homogenized in Trizol reagent (Invitrogen, weight/volume ratio, 1:10). Total RNA was extracted and precipitated by chloroform/isopropanol/ethanol method, treated with DNase I (Ambion, Austin, TX) to remove contaminating DNA, and assessed by spectrophotometry for purity (OD_260_/OD_280_ ratio > 1.8) and amount (OD_260_ of 1 = 40 μg/mL) [42]. First strand cDNA was synthesized by using 2 μg total RNA and iScript^™^ cDNA synthesis kit (170–8891, Bio-Rad), and stored in 100-μL nuclease free dH_2_O. Real time qPCR was performed in a 20 μL reaction containing 2 μL cDNA, 10 μL SYBR green master mix (170–8882; Bio- Rad), and 20 μM of the gene-specific oligonucleotides listed in S1 Table. The thermal cycling conditions were 95°C for 3 min and 40 cycles of 95°C for 15 sec and 60°C for 30 sec. The PCR base line subtracted curve fit mode was applied for determining the threshold cycle (*C*t) by using iCycler iQ real-time detection system software (Bio-Rad). For each target gene, *C*t values were normalized to the mean Ct value for murine *Gapdh* reference cDNA. The relative expression level of target gene was calculated by following the 2^−ΔCt^ [2 ^(-ΔCt sample)^ / 2 ^(– ΔCt of control)^] method [43].

### Size, distribution and molecular characterization of Ev

To quantify size and distribution, Ev purified from different samples were subjected to Nanoparticle Tracking Analysis (NTA) by using PMX-120-12B R2 ZetaView (Particle Metrix, Meerbusch, Germany). Briefly, the Brownian motion of each Ev particle was visualized by a laser light scattering method (at 488 nm) and tracked over 30–45 sec to calculate particle size and concentration. Each measurement was performed for two cycles, scanning 11 cell positions and capturing 60 frames per position per cycle with camera sensitivity 90 volt/μ joule/cm^2^, shutter time 70 milli-sec. The videos were analyzed by ZetaView Software 8.05.05. SP2. Ev samples were diluted 1:10–1:20 in 1X PBS to ensure that the concentration and size distribution of Ev in each sample was optimal for ZetaView analysis (range: 30–60 particles/frame, 15–2000 nm size).

To determine the origin of Ev_DNA,_ real time qPCR and traditional PCR were performed. Real time qPCR was performed as described above using 2 μL of Ev_DNA_ and oligonucleotides pairs to amplify murine (*COII*, *Cytb*, *Gapdh*) and *T*. *cruzi* (*Tc18SrDNA*) DNA sequences. Traditional PCR was performed in a 25 μL reaction containing 2.5 μL of Ev_DNA_, 12.5 μL Go Taq Green master mix (M7122, Promega) and 20 μM of *T*. *cruzi kDNA* (kinetoplast DNA, minicircle)-specific oligonucleotides. The cycling program included an initial denaturation at 95°C for 2 min, 40 cycles of 95°C for 30 sec, 57°C for 30 sec, 72°C for 30 sec, and a final extension at 72°C for 5 min. All oligonucleotides used for qPCR and traditional PCR are listed in S1 Table. The Ev_DNA_ fractions and qPCR and traditional PCR products were resolved on 1.5% agarose gels, stained with 1 μg/mL of ethidium bromide and imaged using Fluor Chem HD2 UV transilluminator (Protein Simple, San Jose, CA).

The levels of 8-hydroxy-2’-deoxy guanosine (8-OHdG, ubiquitous marker of oxidative DNA damage) in Ev_DNA_ fractions from normal and infected cells and mice (WT and *Parp1*^-/-^) were measured by using an 8-OHdG DNA Damage ELISA kit (STA320, Cell Biolabs, San Diego, CA). For this, Ev_DNA_ samples (1 mg/mL) were denatured at 95°C for 5 min and digested with nuclease P1 at 37°C for 2 h to form nucleosides. Samples were then treated with 5U alkaline phosphatase for 1 h at 37°C, centrifuged at 6000 g for 5 min, and supernatants containing Ev_DNA_ fragments were used in an ELISA. Then, 50 μL of supernatant containing Ev_DNA_ fragments were added in triplicate to 96-well plates, and plates were incubated at room temperature for 1 h each with 50 μL of anti-8-OHdG antibody (1:500 dilution) and 100 μL of HRP-conjugated secondary antibody (1:1000 dilution). The color was developed with TMB substrate and change in absorbance was recorded at 450 nm by using a Spectra Max M2 microplate reader (Molecular Devices, Sunnyvale, CA). Standard curve was prepared by using 8-OHdG (100 pg– 20 ng/ mL).

To examine the protein content, Ev samples were subjected to protein extraction with 1X RIPA buffer. Ev and Ev_protein_ fractions (10 μg) were electrophoresed on a 10% polyacrylamide gel by using a Mini-PROTEAN electrophoresis chamber (Bio-Rad). Gels were stained with Coomassie blue and imaged using an Image Quant LAS4000 system (GE Healthcare, Pittsburgh, MA). For Western blotting, proteins were transferred to PVDF membrane using a Criterion Trans-blot System (Bio-Rad) and membranes were blocked for 2 h with 20 mM Tris-HCl (pH 7.4), 136 mM NaCl, 0.1% Tween 20 (TBST) containing 0.5% BSA. Membranes were incubated overnight at 4°C with primary antibody to Mφ markers CD11b (ab133357, 1: 1000 dilution) and CD68 (ab31630, 1:1000 dilution), GAPDH (ab9485, 1:2500 dilution, loading control) and polyclonal sera (1: 50 dilution) from chronically infected mice. All antibodies were purchased from Abcam, MA, USA; and dilutions were made in TBST-0.5% BSA. Membranes were washed with TBST (three times at each step) and incubated for 1 h with HRP-conjugated secondary antibody (1:5000 dilution, Southern Biotech, Birmingham AL). Color was developed by pierce ECL western blot substrate, images were acquired as above, intensity analysis of protein bands was performed by using Image J software (NIH, Bethesda, MD).

### Transfection and NFκB activity by dual luciferase assay

Transfection and dual luciferase assays were conducted by using a Transfection Collection NFκB Transient Pack (79268, BPS Biosciences, San Diego, CA). Briefly, Raw Mφ (30,000 cells/100 μL BPS medium) were seeded in 96-well, clear bottom, tissue culture plates, and allowed to adhere for 24 h. For transfection, 1 μL of NFκB reporter (consists NFκB reporter vector + constitutively expressing Renilla luciferase vector) or negative control reporter (non-inducible luciferase vector + Renilla luciferase vector) were diluted in 15 μL of Opti MEM I medium, mixed with 0.35 μL of Lipofectamine 2000, and added to each well [19]. After incubation for 24 h at 37°C / 5% CO_2_, cells were replenished with fresh BPS medium. Then cells were loaded with Ev isolated from supernatants of normal and infected Mφ or from plasma of non-infected and chronically infected WT and *Parp1*^-/-^ mice. Cells were incubated with Ev for 3 h in the presence or absence of 5 μM and 10 μM of iniparib (PARP1 inhibitor) and/or PF-06928215 (cGAS inhibitor). To measure luciferase activity, equal volumes of firefly luciferase followed by renilla luciferase working solution provided in the Dual Luciferase Assay System (BPS Biosciences) were added, and the release of luminescence was recorded by using a Glomax 96 microplate luminometer (Promega, Madison, WI). The relative luminescence for NFκB reporter (firefly luciferase) was normalized to renilla luciferase (determines transfection efficiency).

### Histology

Heart tissue sections of chronically infected WT and *Parp1*^-/-^ mice were fixed in 10% buffered formalin for 24 h, dehydrated in absolute alcohol, cleared in xylene, and embedded in paraffin. Paraffin-embedded 5-micron tissue-sections were stained with hematoxylin and eosin (H&E) and evaluated by light microscopy. Tissue section slides (three mice per group, at least two slides per tissue, ten microscopic fields per slide) were analyzed by light microscopy, and the presence of inflammatory cells was scored as (0)—absent/none, (1)—focal or mild with ≤ 1 foci, (2)—moderate with ≥ 2 inflammatory foci, (3)—extensive with generalized coalescing of inflammatory foci or disseminated inflammation, and (4)—severe with diffused inflammation, interstitial edema, and loss of tissue integrity [44].

### Immunohistochemistry

To visualize *in situ* population of macrophages, paraffin-embedded 5 μm heart tissue sections were deparaffinized, suspended in 0.01 M sodium citrate buffer (pH 6.0) and incubated for 10 min in a boiling water bath to unmask the antigens, and marked with an ImmEdge hydrophobic barrier pen (Vector laboratories). Slides were then incubated with Bloxall blocking solution (Immpress duet, Vector Laboratories) for 10 min to quench endogenous peroxidase activity and with 2.5% normal horse serum for 20 minutes to block non-specific antibody binding. Next, tissues were incubated for 6 h– 18 h with primary antibodies diluted in 1X PBS containing 1% BSA and 0.1% Triton X-100. These included rabbit monoclonal anti-CD11b (ab133357, Abcam, 1: 1000 dilution), mouse monoclonal anti-CD68 (ab31630, Abcam, 1: 100 dilution), mouse monoclonal anti-CD206 (sc58986, Santa Cruz Biotech, 1: 50 dilution), mouse monoclonal anti-TNF-α (sc52746, Santa Cruz Biotech, 1: 50 dilution), rabbit polyclonal anti-IL-10 (ab192271, Abcam, 1: 500 dilution), and mouse monoclonal prediluted anti-CD11b (ab75693, Abcam) antibodies. Slides were washed in 1X PBS and incubated with ImmPRESS Duet Double Detection Reagent (MP-7714, Vector laboratories) containing horseradish peroxidase (HRP)-conjugated horse anti-rabbit IgG and alkaline phosphatase (AP)-conjugated horse anti-mouse IgG antibodies. Subsequently, tissue sections were sequentially stained with DAB EqV HRP substrate (brown color) and Immpact vector red AP substrate (magenta color). Finally, slides were rinsed in 1X PBS buffer and mounted in VectaMount AQ Aqueous Mounting Medium (H- 5501, Vector Laboratories) [45].

All slides were imaged at 20X and 60X magnification by light microscopy by using an Olympus BX-15 microscope (Center Valley, PA) equipped with digital camera and Simple PCI software (v.6.0, Compix, Sewickley, PA). Tissue section slides (n = 3 mice per group, at least two slides per tissue) were analyzed in nine microscopic fields (mf), and immuno-stained areas were scored as (0) = < 10%, (1^+^) = 10–25%, (2^+^) = 25–50%, (3^+^) = 50–75%, and (4^+^) = > 75% of scanned area. The intensity of staining was scored as (1)—weak, (2)—moderate, and (3), strong. Finally, quick histology score was calculated by multiplying the score for the immuno-stained area with the score for staining intensity.

### Statistical analysis

All experiments were repeated at least twice. In general, *in vitro* experiments were conducted with duplicate or triplicate biological replicates per group with two or three observations per sample per experiment. Murine samples (n = 10 per group for Ev analysis and n = 3 per group for histology studies) were analyzed in duplicate. All data were analyzed by using an InStat version 5 (GraphPad, La Jolla, CA) software. Mean values were compared by unpaired Student’s two tailed t-test (for comparison of two groups) and one-way analysis of variance (ANOVA) with *post hoc* correction test (for comparison of multiple groups). Data are presented as mean ± standard deviation (SD). A p value of < 0.05 was considered as minimum level of significance for the comparison of minimum two variables.

## Supporting information

S1 Fig **(A) *T*. *cruzi* induces macrophage (M**φ) **release of proinflammatory extracellular vesicles (Ev) at 72 h**. RAW 264.7 Mφ were infected with *T*. *cruzi* (cell: parasite ratio, 1:3) and supernatants were used to isolate *T*. *cruzi-*induced extracellular vesicles (TEv) at 24, 48, and 72 h. Next, cultured Mφ were incubated with TEv for 48 h, and TNF-α release was measured by an ELISA. **(B) Fetal bovine serum has no effect on TEv signaling of M**φ **response**. Cultured Raw Mφ were incubated with TEv (± 10% heat inactivated FBS) for 6, 18, and 48 h, and TNF-α release was measured.
(TIF)

S2 Fig Ev released by *T*. *cruzi* elicit proinflammatory response. *T*. *cruzi* trypomastigotes were incubated in complete medium for 72 h, and *Tc*Ev shed in the medium were isolated. Cultured Mφ were incubated with *T*cEv or *Tc*-induced Ev isolated from infected Mφ and release of TNF-α **(A)** and IL-6 **(B)** was monitored by an ELISA. Mφ incubated with live *T*. *cruzi* trypomastigotes (cell: parasite ratio, 1:3) and IFN-γ (20 ng/mL) were used as positive controls. Statistical significance is presented as ^&&&^ p≤ 0.001 (*T*. *cruzi* infection vs. *Tc*Ev).
(TIF)

S3 Fig **(A-C) TEv induced proinflammatory gene expression in murine bone marrow derived WT and *Parp1-/-* Mφ at 18 h post-incubation**. RAW 264.7 Mφ were incubated with media only or *T*. *cruzi* (cell: parasite ratio, 1:3) for 72 h, and supernatants were used to isolate normal (NEv) and *T*. *cruzi-*induced (TEv) extracellular vesicles, respectively. Bone marrow cells of WT or *Parp1*-/- mice were matured into primary Mφ as described in Materials and Methods. Next, primary BM-Mφ were incubated with NEv_Raw_ or TEv_Raw_ in presence or absence of 20 ng/mL IFN-γ for 18 h and cytokines’ gene expression was evaluated by RT-qPCR. Primary BM-M**φ** incubated with *T*. *cruzi* and IFN-γ were used as controls. **(D-F) M**φ **activation by Ev induced in *T*. *cruzi* infected non-immune cells (± PARP1 inhibitor)**. C2C12 muscle cells were incubated with media only or *T*. *cruzi* (cell: parasite ratio, 1: 3) for 72 h, and Ev were isolated from supernatants of normal (NEv) and *Tc*-infected (TEv) cells. Next, Raw Mφ were incubated with NEv_C2C12_ or TEv_C2C12_ in presence or absence of 20 ng/mL IFN-γ and 5 μM iniparib (inib, selective PARP1 inhibitor) for 48 h, and release of TNF-α, IL-6, and IL-1β cytokines was monitored by an ELISA. Mφ incubated with *T*. *cruzi* and IFN-γ (± iniparib) were used as controls. Data are representative of ≥ 2 independent experiments (2–3 biological replicates per treatment, and 2–3 observations per sample) and presented as mean ± SD. Horizontal bar indicates the compared groups. Statistical significance is captured with + NEv vs. TEv, *effect of IFN-γ on TEv, and ^*i*^ effect of Parp1 knockdown on TEv+IFN-γ. The p values of ≤ 0.05, ≤ 0.01, and ≤ 0.001 are presented by one, two, and three symbol characters, respectively. Horizontal bar indicates the compared groups.
(TIF)

S4 Fig Characterization of Ev_DNA_. Total DNA was isolated from NEv and TEv samples. **(A-F)** Bar graphs show real-time qPCR amplification of murine *COII* ***(A-C)*** and *Cytb* ***(D-F)*** DNA sequences in NEv and TEv of non-infected and infected Raw Mφ ***(A&D)***, and WT ***(B&E)*** and *Parp1*^*-/-*^ ***(C&F)*** mice that were non-infected or acutely (ac) and chronically (ch) infected with *T*. *cruzi*. Data are presented as fold change ± SD and normalized to *mGapdh* (two biological replicates each with triplicate observations per sample for A & D, and n = 5 for B, C, E & F). **(G-I)** Representative gel images show the amplification of single bands for *COII*, *Cytb*, *and Gapdh*.
(TIF)

S5 Fig Myocardial CD 11b^+^ macrophage profile in Chagas disease (± PARP1). Mice (WT and *Parp1*^*-/-*^) were euthanized at 150 days post-infection corresponding to chronic disease phase. Myocardial tissue sections of non-infected and infected mice were subjected to immunohistochemistry staining. Shown is the myocardial expression of CD11b, presented as semi-quantitative immunohistochemistry quick score ± SD (n = 3 mice per group, two tissue sections per mouse, 9 microscopic fields per tissue section, 20X magnification). Significance is annotated as ^+++^ infected vs. non-infected (p<0.001) and ** WT.*Tc* vs. *Parp1*^*-/-*^.*Tc* (p<0.01).
(TIF)

S1 Table Oligonucleotides used in this study. (DOCX)
