# Supplementary figures and images for: PARP1-cGAS-NF-κB pathway of proinflammatory macrophage activation by extracellular vesicles released during Trypanosoma cruzi infection and Chagas disease
Source: PLoS Pathog. 2020 Apr 21;16(4):e1008474. doi: 10.1371/journal.ppat.1008474 (PMC7173744; doi:10.1371/journal.ppat.1008474)

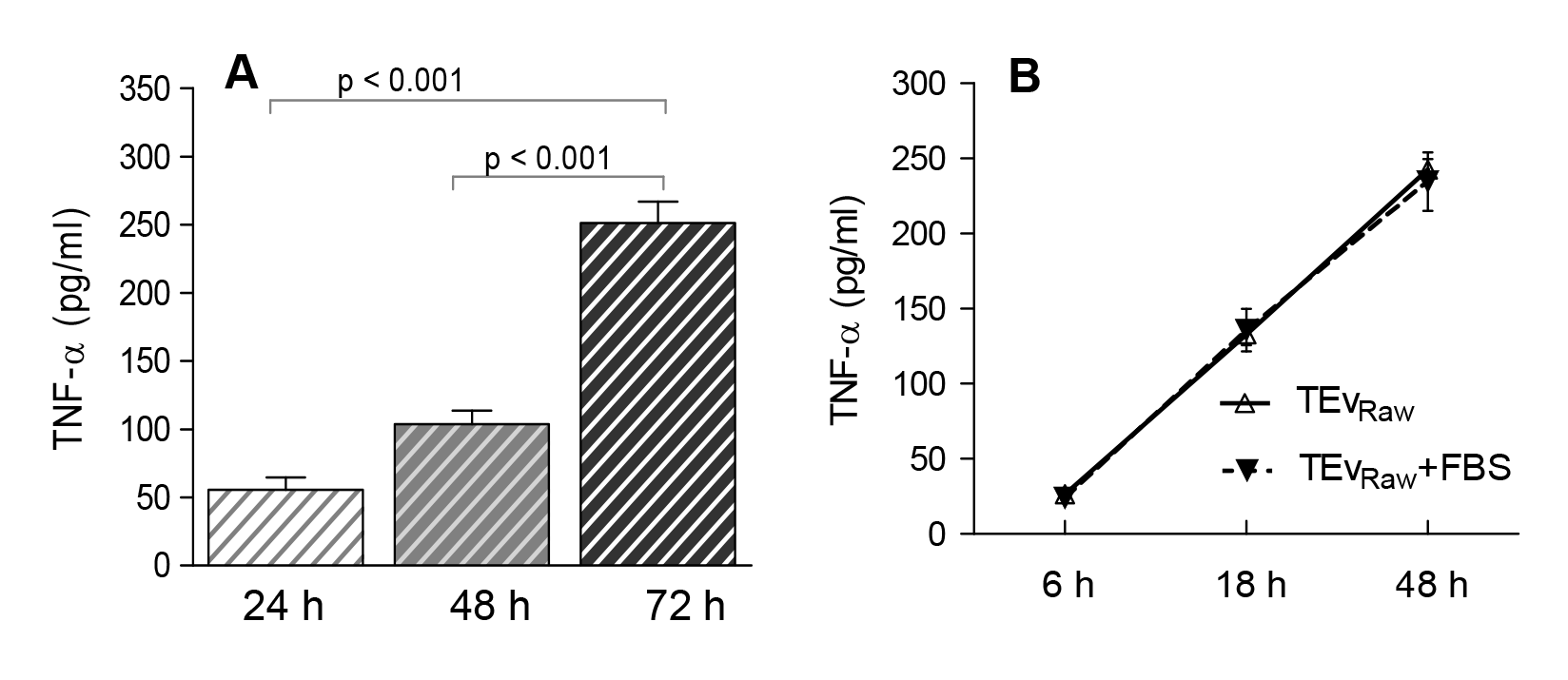

Supplement: S1 Fig — (A) T. cruzi induces macrophage (Mφ) release of proinflammatory extracellular vesicles (Ev) at 72 h. RAW 264.7 Mφ were infected with T. cruzi (cell: parasite ratio, 1:3) and supernatants were used to isolate T. cruzi-induced extracellular vesicles (TEv) at 24, 48, and 72 h. Next, cultured Mφ were incubated with TEv for 48 h, and TNF-α release was measured by an ELISA. (B) Fetal bovine serum has no effect on TEv signaling of Mφ response. Cultured Raw Mφ were incubated with TEv (± 10% heat inactivated FBS) for 6, 18, and 48 h, and TNF-α release was measured. (TIF) [file ppat.1008474.s001.tif]

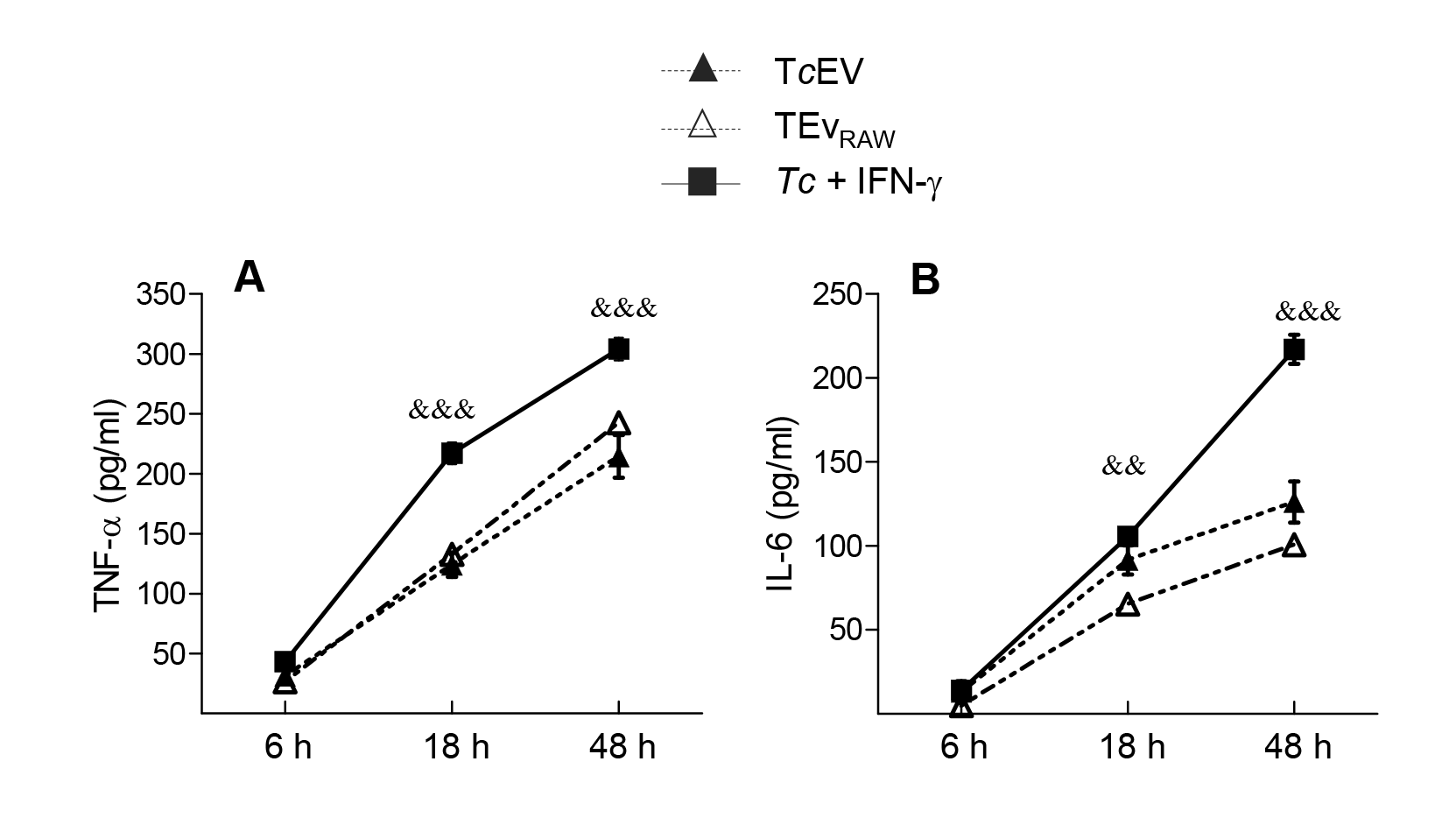

Supplement: S2 Fig — T. cruzi trypomastigotes were incubated in complete medium for 72 h, and TcEv shed in the medium were isolated. Cultured Mφ were incubated with TcEv or Tc-induced Ev isolated from infected Mφ and release of TNF-α (A) and IL-6 (B) was monitored by an ELISA. Mφ incubated with live T. cruzi trypomastigotes (cell: parasite ratio, 1:3) and IFN-γ (20 ng/mL) were used as positive controls. Statistical significance is presented as &&& p≤ 0.001 (T. cruzi infection vs. TcEv). (TIF) [file ppat.1008474.s002.tif]

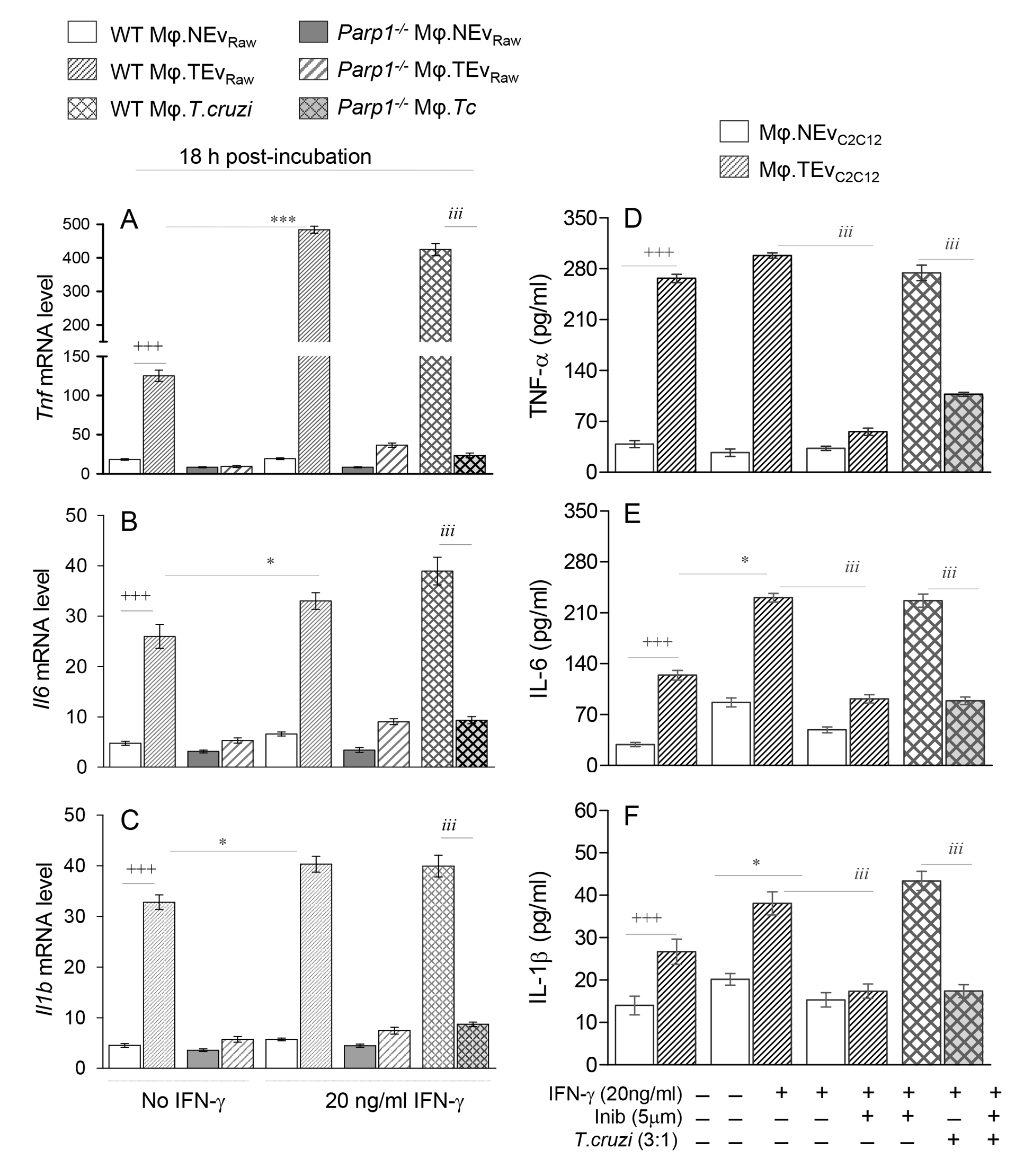

Supplement: S3 Fig — (A-C) TEv induced proinflammatory gene expression in murine bone marrow derived WT and Parp1-/- Mφ at 18 h post-incubation. RAW 264.7 Mφ were incubated with media only or T. cruzi (cell: parasite ratio, 1:3) for 72 h, and supernatants were used to isolate normal (NEv) and T. cruzi-induced (TEv) extracellular vesicles, respectively. Bone marrow cells of WT or Parp1-/- mice were matured into primary Mφ as described in Materials and Methods. Next, primary BM-Mφ were incubated with NEvRaw or TEvRaw in presence or absence of 20 ng/mL IFN-γ for 18 h and cytokines’ gene expression was evaluated by RT-qPCR. Primary BM-Mφ incubated with T. cruzi and IFN-γ were used as controls. (D-F) Mφ activation by Ev induced in T. cruzi infected non-immune cells (± PARP1 inhibitor). C2C12 muscle cells were incubated with media only or T. cruzi (cell: parasite ratio, 1: 3) for 72 h, and Ev were isolated from supernatants of normal (NEv) and Tc-infected (TEv) cells. Next, Raw Mφ were incubated with NEvC2C12 or TEvC2C12 in presence or absence of 20 ng/mL IFN-γ and 5 μM iniparib (inib, selective PARP1 inhibitor) for 48 h, and release of TNF-α, IL-6, and IL-1β cytokines was monitored by an ELISA. Mφ incubated with T. cruzi and IFN-γ (± iniparib) were used as controls. Data are representative of ≥ 2 independent experiments (2–3 biological replicates per treatment, and 2–3 observations per sample) and presented as mean ± SD. Horizontal bar indicates the compared groups. Statistical significance is captured with + NEv vs. TEv, *effect of IFN-γ on TEv, and i effect of Parp1 knockdown on TEv+IFN-γ. The p values of ≤ 0.05, ≤ 0.01, and ≤ 0.001 are presented by one, two, and three symbol characters, respectively. Horizontal bar indicates the compared groups. (TIF) [file ppat.1008474.s003.tif]

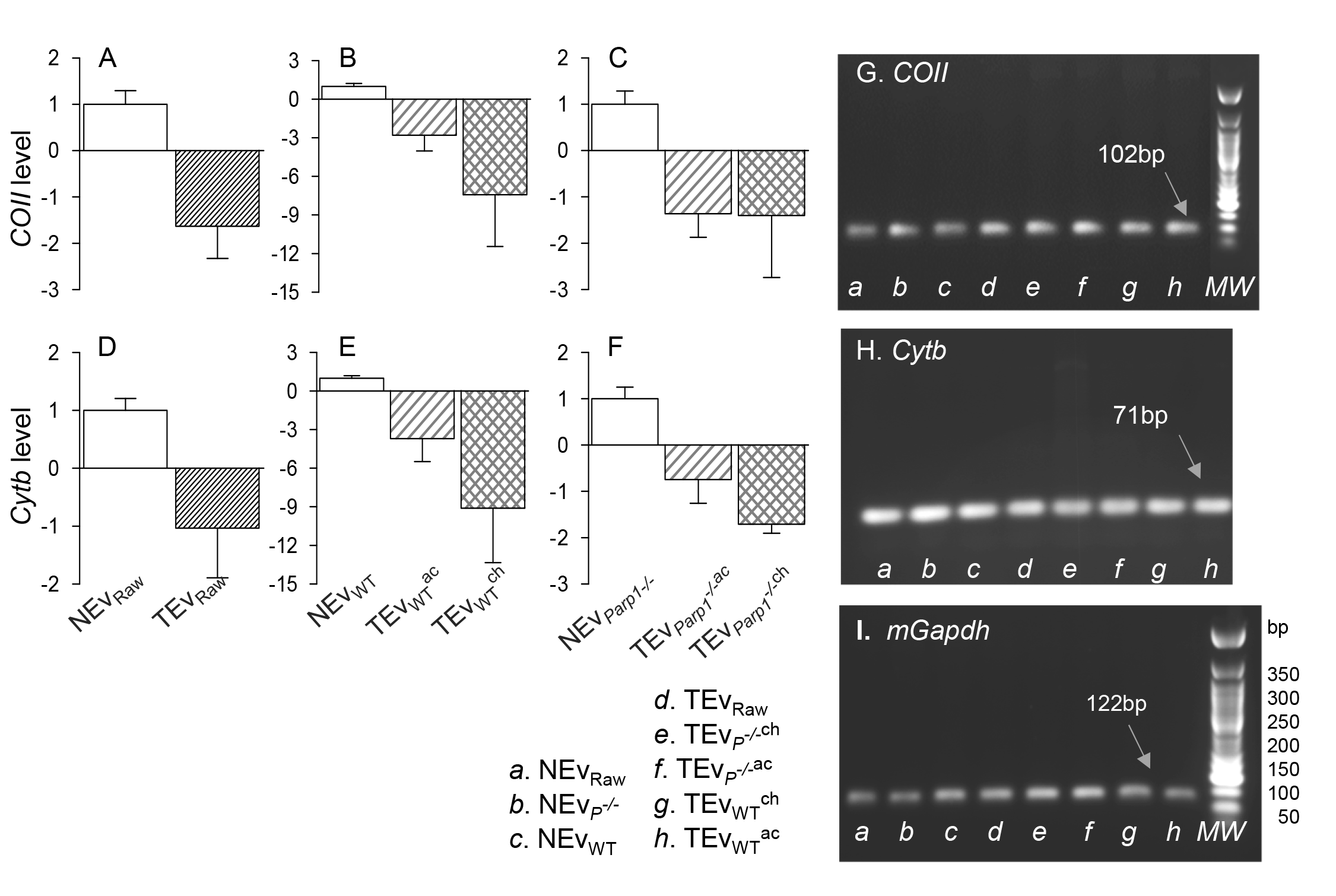

Supplement: S4 Fig — Total DNA was isolated from NEv and TEv samples. (A-F) Bar graphs show real-time qPCR amplification of murine COII (A-C) and Cytb (D-F) DNA sequences in NEv and TEv of non-infected and infected Raw Mφ (A&D), and WT (B&E) and Parp1-/- (C&F) mice that were non-infected or acutely (ac) and chronically (ch) infected with T. cruzi. Data are presented as fold change ± SD and normalized to mGapdh (two biological replicates each with triplicate observations per sample for A & D, and n = 5 for B, C, E & F). (G-I) Representative gel images show the amplification of single bands for COII, Cytb, and Gapdh. (TIF) [file ppat.1008474.s004.tif]

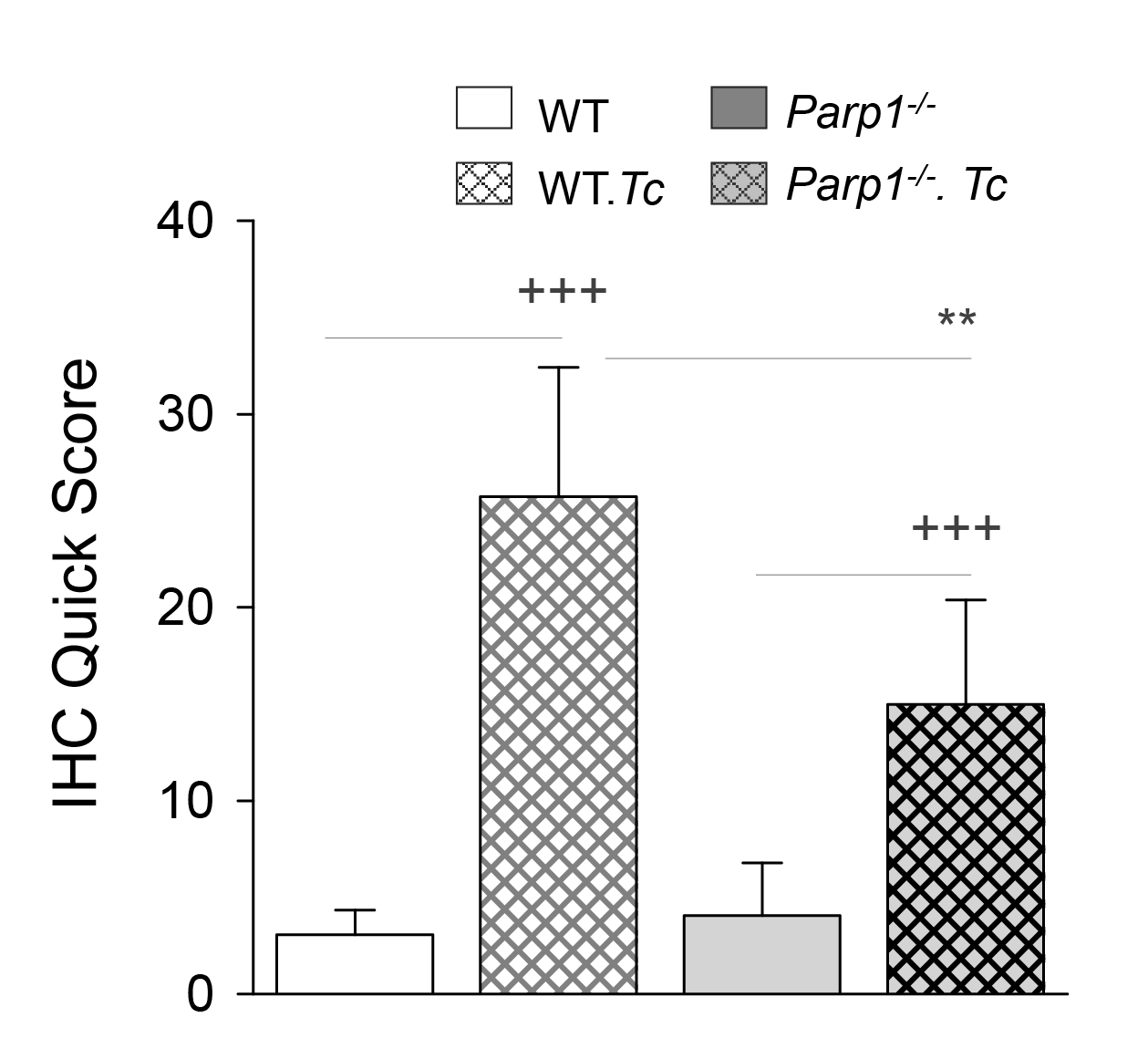

Supplement: S5 Fig — Mice (WT and Parp1-/-) were euthanized at 150 days post-infection corresponding to chronic disease phase. Myocardial tissue sections of non-infected and infected mice were subjected to immunohistochemistry staining. Shown is the myocardial expression of CD11b, presented as semi-quantitative immunohistochemistry quick score ± SD (n = 3 mice per group, two tissue sections per mouse, 9 microscopic fields per tissue section, 20X magnification). Significance is annotated as +++ infected vs. non-infected (p<0.001) and ** WT.Tc vs. Parp1-/-.Tc (p<0.01). (TIF) [file ppat.1008474.s005.tif]
