# Supplementary material for: PARP1-cGAS-NF-κB pathway of proinflammatory macrophage activation by extracellular vesicles released during Trypanosoma cruzi infection and Chagas disease
Source: PLoS Pathog. 2020 Apr 21;16(4):e1008474. doi: 10.1371/journal.ppat.1008474 (PMC7173744; doi:10.1371/journal.ppat.1008474)
Supplement: S1 Table — (DOCX) [file ppat.1008474.s006.docx]

| **S1 Table: Oligonucleotides used in this study** | | | | |
| --- | --- | --- | --- | --- |
| **Gene name** | **Acc #** | **5’-3’ Forward** | **5’-3’ Reverse** | **Size (bp)** |
| **Oligonucleotides used for RTqPCR** | | | | |
| *mIl1b* | NM_008361 | TGCCACCTTTTGACAGTGATG | TGATGTGCTGCTGCGAGATT | 351 |
| *mIl6* | NM_031168 | ATGGATGCTACCAAACTGGAT | TGAAGGACTCTGGCTTTGTCT | 139 |
| *mTnf* | NM_013693 | CCACTGTGGGAAGCTGTCTT | AAGCAAAAGAGGAGGCAACA | 118 |
| *mParp1* | NM_007415 | GATCCCATCGACGTCAACTAC | GATCACTTCCAGGTCATAGGC | 141 |
| *mGapdh* | NM_001289726.1 | AACTTTGGCATTGTGGAAGG | ACACATTGGGGGTAGGAACA | 223 |
| **Oligonucleotides used for qPCR or traditional PCR** | | | | |
| *mCOII* | NC_012387 | ATTGCCCTCCCCTCTCTACGCA | CGTAGCTTCAGTATCATTGGTGCC | 102 |
| m*Cytb* | NC_010339.1 | GCAACCTTGACCCGATTCTTCGC | TGAACGATTGCTAGGGCCGCG | 71 |
| *T.cruzi kDNA*_conserved_ | X04680 | AAATAATGTACGGGKGAGATGCA | GGTTCGATTGGGGTTGGTGTAATATA | 330 |
| *T.cruzi*  *kDNA*_variable_ | X04680 | ATAATGTACGGGKGAGATGCATG | GGGTTCGATTGGGGTTGGTGT | 320 |
| *Tc18SrDNA* | [NC_018331.1](http://www.ncbi.nlm.nih.gov/nucleotide/401424696?from=982402&to=991477&report=gbwithparts) | TTTT GGGC AACA GCAG GTCT | CTGC GCCT ACGA GACA TTCC | 200 |
| *mGapdh* | NC_000072.6 | ACAATTTCCATCCCAGACCC | CCCAACACCGCATTAAAACC | 122 |
